# Biallelic *MAD2L1BP* (p31^comet^) mutation is associated with mosaic aneuploidy and juvenile granulosa cell tumors

**DOI:** 10.1172/jci.insight.170079

**Published:** 2023-11-22

**Authors:** Ghada M. H. Abdel-Salam, Susanne Hellmuth, Elise Gradhand, Stephan Käseberg, Jennifer Winter, Ann-Sophie Pabst, Maha M. Eid, Holger Thiele, Peter Nürnberg, Birgit S. Budde, Mohammad Reza Toliat, Ines B. Brecht, Christopher Schroeder, Axel Gschwind, Stephan Ossowski, Friederike Häuser, Heidi Rossmann, Mohamed S. Abdel-Hamid, Ibrahim Hegazy, Ahmed G. Mohamed, Dominik T. Schneider, Aida Bertoli-Avella, Peter Bauer, Jillian N. Pearring, Rolph Pfundt, Alexander Hoischen, Christian Gilissen, Dennis Strand, Ulrich Zechner, Soha A. Tashkandi, Eissa A. Faqeih, Olaf Stemmann, Susanne Strand, Hanno J. Bolz

**Affiliations:** 1 Department of Clinical Genetics, Human Genetics and Genome Research Institute, National Research Centre, Cairo, Egypt.; 2 Chair of Genetics, University of Bayreuth, Bayreuth, Germany.; 3 Senckenberg Institute of Pathology, University Hospital Frankfurt, Frankfurt, Germany.; 4 Institute of Human Genetics, University Medical Center Mainz, Mainz, Germany.; 5 Human Cytogenetics Department, Human Genetics and Genome Research Institute, National Research Centre, Cairo, Egypt.; 6 Cologne Center for Genomics and; 7 Center for Molecular Medicine Cologne, University Hospital of Cologne, University of Cologne, Cologne, Germany.; 8 Cologne Excellence Cluster on Cellular Stress Responses in Aging-Associated Diseases (CECAD), University of Cologne, Cologne, Germany.; 9 Paediatric Haematology/Oncology, Department of Paediatrics, University Hospital Tübingen, Tübingen, Germany.; 10 Institute of Medical Genetics and Applied Genomics, Eberhard-Karls University, Tübingen, Germany.; 11 Institute of Clinical Chemistry and Laboratory Medicine, University Medical Center Mainz, Mainz, Germany.; 12 Medical Molecular Department, Human Genetics and Genome Research Institute, National Research Centre, Cairo, Egypt.; 13 Pediatrics Department, Faculty of Medicine, Cairo University, Cairo, Egypt.; 14 Clinic of Pediatrics, University Witten/Herdecke, Dortmund, Germany.; 15 CENTOGENE GmbH, Rostock, Germany.; 16 Department of Ophthalmology and Visual Sciences and; 17 Department of Cell and Developmental Biology, University of Michigan School of Medicine, Ann Arbor, Michigan, USA.; 18 Department of Human Genetics and Radboud Institute for Molecular Life Sciences and; 19 Department of Internal Medicine, Radboud University Medical Center, Nijmegen, Netherlands.; 20 Department of Internal Medicine I, University Medical Center Mainz, Mainz, Germany.; 21 Senckenberg Centre for Human Genetics, Frankfurt am Main, Germany.; 22 Cytogenetics Laboratory, Pathology and Clinical Laboratory Medicine Administration (PCLMA), King Fahad Medical City, Second Central Healthcare Cluster (C2), Riyadh, Saudi Arabia.; 23 Section of Medical Genetics, Children’s Specialist Hospital, King Fahad Medical City, Riyadh, Saudi Arabia.; 24 Institute of Human Genetics, University Hospital of Cologne, University of Cologne, Cologne, Germany.

**Keywords:** Genetics, Oncology, Cell cycle, Genetic diseases, Genetic instability

## Abstract

*MAD2L1BP*-encoded p31^comet^ mediates Trip13-dependent disassembly of Mad2- and Rev7-containing complexes and, through this antagonism, promotes timely spindle assembly checkpoint (SAC) silencing, faithful chromosome segregation, insulin signaling, and homology-directed repair (HDR) of DNA double-strand breaks. We identified a homozygous *MAD2L1BP* nonsense variant, R253*, in 2 siblings with microcephaly, epileptic encephalopathy, and juvenile granulosa cell tumors of ovary and testis. Patient-derived cells exhibited high-grade mosaic variegated aneuploidy, slowed-down proliferation, and instability of truncated p31^comet^ mRNA and protein. Corresponding recombinant p31^comet^ was defective in Trip13, Mad2, and Rev7 binding and unable to support SAC silencing or HDR. Furthermore, C-terminal truncation abrogated an identified interaction of p31^comet^ with tp53. Another homozygous truncation, R227*, detected in an early-deceased patient with low-level aneuploidy, severe epileptic encephalopathy, and frequent blood glucose elevations, likely corresponds to complete loss of function, as in *Mad2l1bp^–/–^* mice. Thus, human mutations of p31^comet^ are linked to aneuploidy and tumor predisposition.

## Graphical abstract

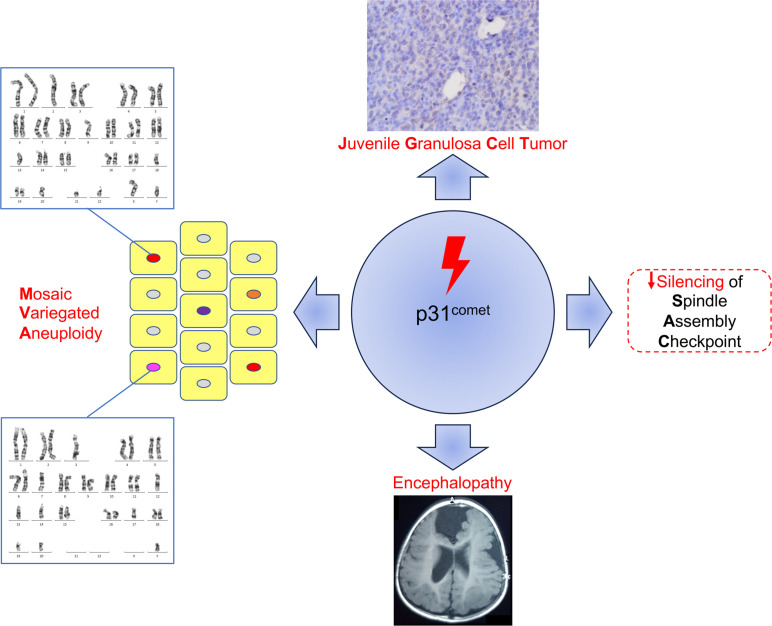

## Introduction

Aneuploidy is a major cause of miscarriage, birth defects, and developmental delay/intellectual disability (1), and it is a hallmark of human cancer (2). Accuracy in chromosome duplication, segregation, and DNA damage repair is crucial for avoiding genomic instability and tumorigenesis (3). Eukaryotic cells rely on checkpoint systems: during interphase, they ensure genome integrity via repair of DNA defects. Before mitosis, the activated spindle assembly checkpoint (SAC) inhibits the anaphase-promoting complex/cyclosome (APC/C), thereby delaying anaphase onset until all chromosomes’ kinetochores are properly attached to spindle microtubules to ensure an equal segregation among daughter cells. Unattached kinetochores convert MAD2 to its CDC20-bound active closed form, and both then associate with BUBR1 (encoded by *BUB1B*) and BUB3 to form an APC/C-inhibitory complex, the mitotic checkpoint complex (MCC). For MCC inactivation, *MAD2L1BP*-encoded p31^comet^ binds and inactivates MAD2 (4), causing disruption of the MCC and promoting timely anaphase onset. Hence, the interplay of p31^comet^, MAD2, and BUB1B is mandatory for proper chromosome segregation and genome stability.

HORMA proteins, like Mad2 and Rev7, typically switch from an inactive “open” into an active “closed” conformational state upon binding to various closure motif-containing partners. In doing so, Mad2 counteracts anaphase onset and insulin signaling, while Rev7 promotes nonhomologous end joining (NHEJ) and translesion synthesis. Although p31^comet^ harbors a HORMA domain itself, it is special in that it exists only in the closed conformation and acts as an antagonist of Mad2 and Rev7. It does so by recruitment of the AAA-ATPase Trip13 to Mad2- and Rev7-containing complexes, thereby mediating their active disassembly and inactivation (5).

We identified a homozygous *MAD2L1BP* pathogenic nonsense variant (R253*) in 2 siblings with microcephaly, brain malformations (with cysts and polymicrogyria), seizures, developmental delay, and juvenile granulosa cell tumor (JGCT) of ovary and testis, respectively. The mutation leads to high-grade mosaic variegated aneuploidy (MVA), instability of p31^comet^, slowed proliferation, and consecutive insensitivity to chemotherapeutic drugs in fibroblasts. Expression of corresponding p31^comet^ constructs revealed impaired interaction with other mitotic checkpoint proteins and with tp53. DNA repair was shifted from homology-directed repair (HDR) toward NHEJ, but no specific mutational signature was found in JGCT DNA. A more N-terminal homozygous nonsense mutation was detected in an early-deceased boy with moderate aneuploidy, but severe brain malformations and refractory seizures verified the association of p31^comet^ with a human MVA-type syndrome.

## Results

### Clinical characterization.

**Figure 1:**
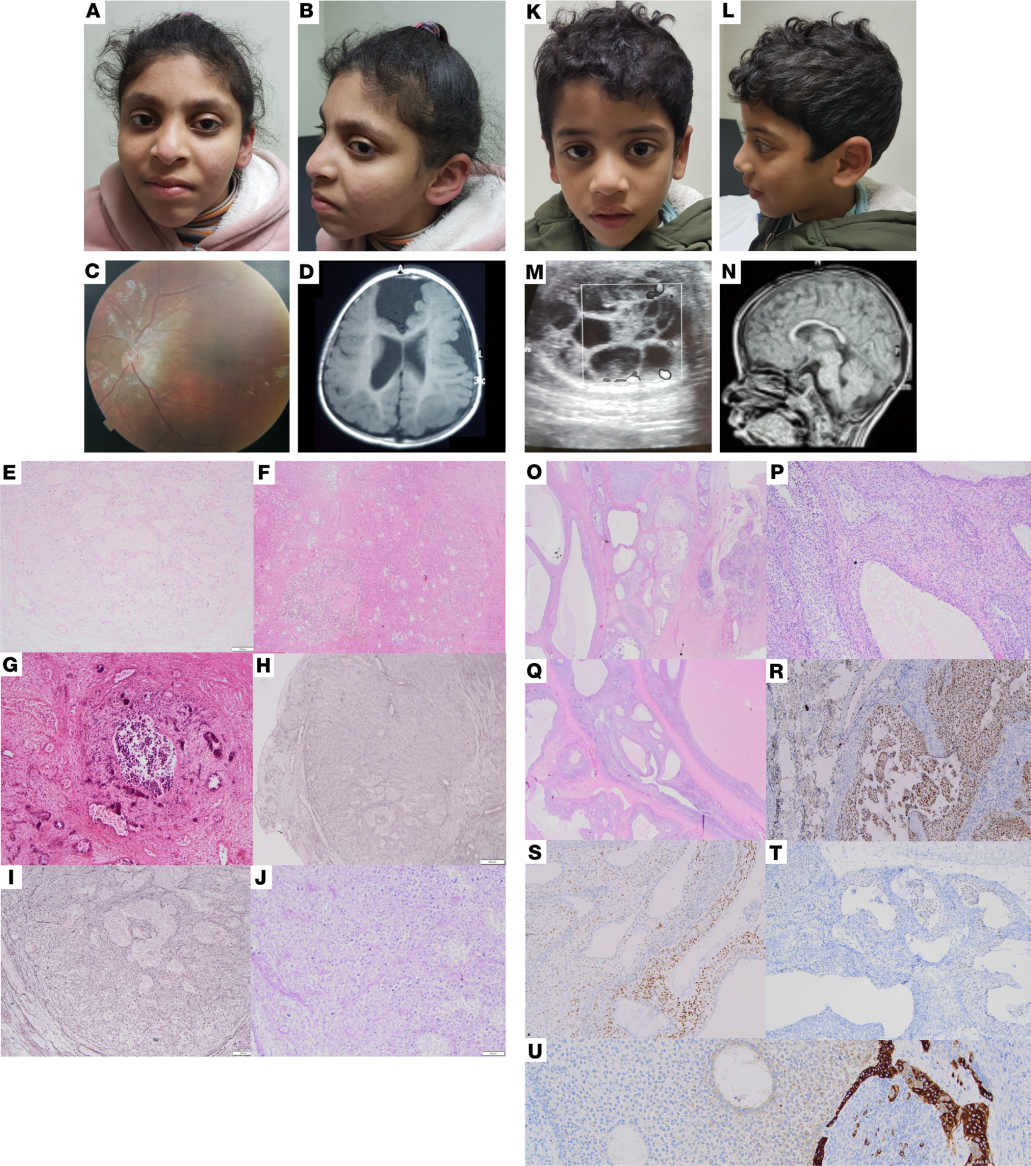
Clinical phenotype of family 1 patients. (**A**–**J**) Patient 1a. (**A** and **B**) Mild facial dysmorphism (4 years): long face, upward slanting sparse outer eyebrows, low columella, pointed chin, and microcephaly. (**C**) Fundus: retinal pigmentation defects. (**D**) Cranial MRI (cMRI) (24 months): underdeveloped brain with dysplastic frontal lobe, interhemispheric cyst, and polymicrogyria on the right near the cyst. Incomplete opercularization and left parietal cyst. (**E**–**J**) Histology of ovarian GCT. (**E**–**G**) H&E staining shows the faint preserved outlines of the macrofollicular architecture. Regressive calcification is visible in **C**. (**H** and **I**) Reticulin stains the preserved fibers, highlighting the typical and unique architecture for JGCT; **E**, at higher magnification. (**J**) Periodic acid–Schiff (PAS) staining shows the typical cytology of the JGCT with monomorphic, round to ovoid cells. (**K**–**U**) Patient 1b. (**K** and **L**) Face with mild dysmorphism (2 years of age). (**M**) Ultrasound image of left testicular mass. (**N**) Thin corpus callosum (cMRI at the age of 18 months). (**O**–**U**) Histology of testicular GCT. (**O**) Testis shows a macrofollicular pattern and contains multiple cysts replacing preexisting prepubertal testicular tissue. (**P**) Multilayered tumor cells surrounded by spindle cell stroma, reminiscent of ovarian follicles. (**Q**) Cysts contain pink mucoid material. The cells of the inner layer are pale and have luteinized cytoplasm. (**R**) Tumor cells show progesterone receptor (PR) expression. (**S**) Tumor cells with diffuse S100 expression. (**T**) Faint expression of estrogen receptor (ER). (**U**) Patchy expression of inhibin. (**E**, **F**, **H**, and **O**) Original magnification, 2×; scale bar: 500 μm. (**G**, **I**, **S**, and **T**) Original magnification, 4×; scale bar: 300 μm. (**J**, **P**, and **Q**) Original magnification, 10×, scale bar: 100 μm. (**R** and **U**) Original magnification, 40×, scale bar: 50 μm.

**Figure 2:**
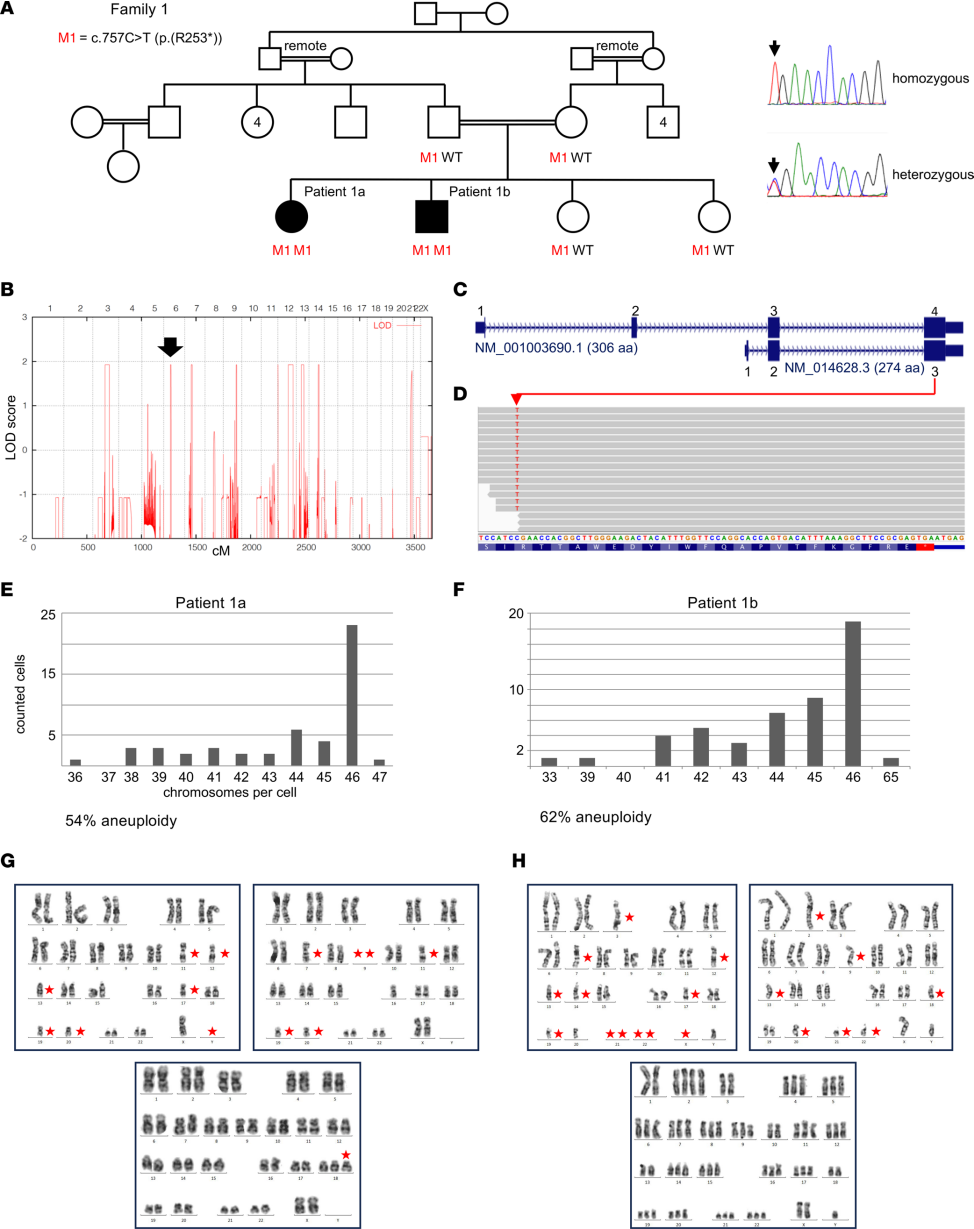
*MAD2L1BP* mutation and MVA in family 1. (**A**) Pedigree. Filled symbols: Homozygous *MAD2L1BP* nonsense variant, c.757C>T [p.(R253*)]; affected. M, mutant allele; WT, wild-type allele. Electropherograms: homozygosity (patient 1a, patient 1b) and heterozygous carriership (healthy sisters, parents). (**B**) Regions with runs of homozygosity identified by genome-wide linkage analysis. Arrow, chromosome 6 region comprising *MAD2L1BP*. (**C**) Scheme of major *MAD2L1BP* isoforms. (**D**) Schematic representation of mapped sequencing reads (forward strand) visualized with the Integrative Genomics Viewer. In the patients, the c.757C>T mutation was present in all respective reads. (**E**) High-grade aneuploidy of cultivated lymphocytes from peripheral blood in patient 1a and (**F**) patient 1b. (**G** and **H**) Representative examples of aberrant karyotypes for both patients, including a 65,XXY constellation (bottom, right). Red asterisks in the karyograms indicate supernumerary or missing chromosomes.

Patient 1a (Figure 1, A–J) was born to consanguineous Egyptian parents (first cousins, family 1; Figure 2A) at term by vaginal delivery after uneventful pregnancy with relatively low birth weight. Nystagmus, abnormal chorioretinal pigmentation, and small optic discs were noted, and visually evoked potentials (VEPs) indicated bilateral delay of retinocortical transmission. At 14 months, she underwent surgery for a JGCT of the left ovary, with subsequent antihormonal therapy (tamoxifen) for 12 months and complete remission since then. cMRI revealed brain cysts, dysplastic frontal lobes, dysgyria/polymicrogyria, arched corpus callosum, and hypoplastic vermis. When attending the genetics clinic at 4 years of age, microcephaly, mild facial dysmorphism, neurological abnormalities (hypotonia, increased reflexes, positive Babinski sign, involuntary head nodding), and developmental delay (IQ: 56) were noted. At 5.5 years of age, she developed generalized tonic-clonic seizures. Metabolic screens and repeated oral glucose tolerance tests were normal in both siblings. Patient 1b (Figure 1, K–U), the brother of patient 1a, was born after uneventful pregnancy with low birth weight (2,700 g; –1.5 SD). At 11 months, radical left orchiectomy was conducted, followed by the diagnosis of JGCT of the testis. He was referred to the genetics clinic at 21 months of age and presented with microcephaly, as well as ophthalmological and neurologic abnormalities similar to patient 1a, but with less pronounced brain abnormalities in the cMRI and with normal psychomotor development.

**Figure 3:**
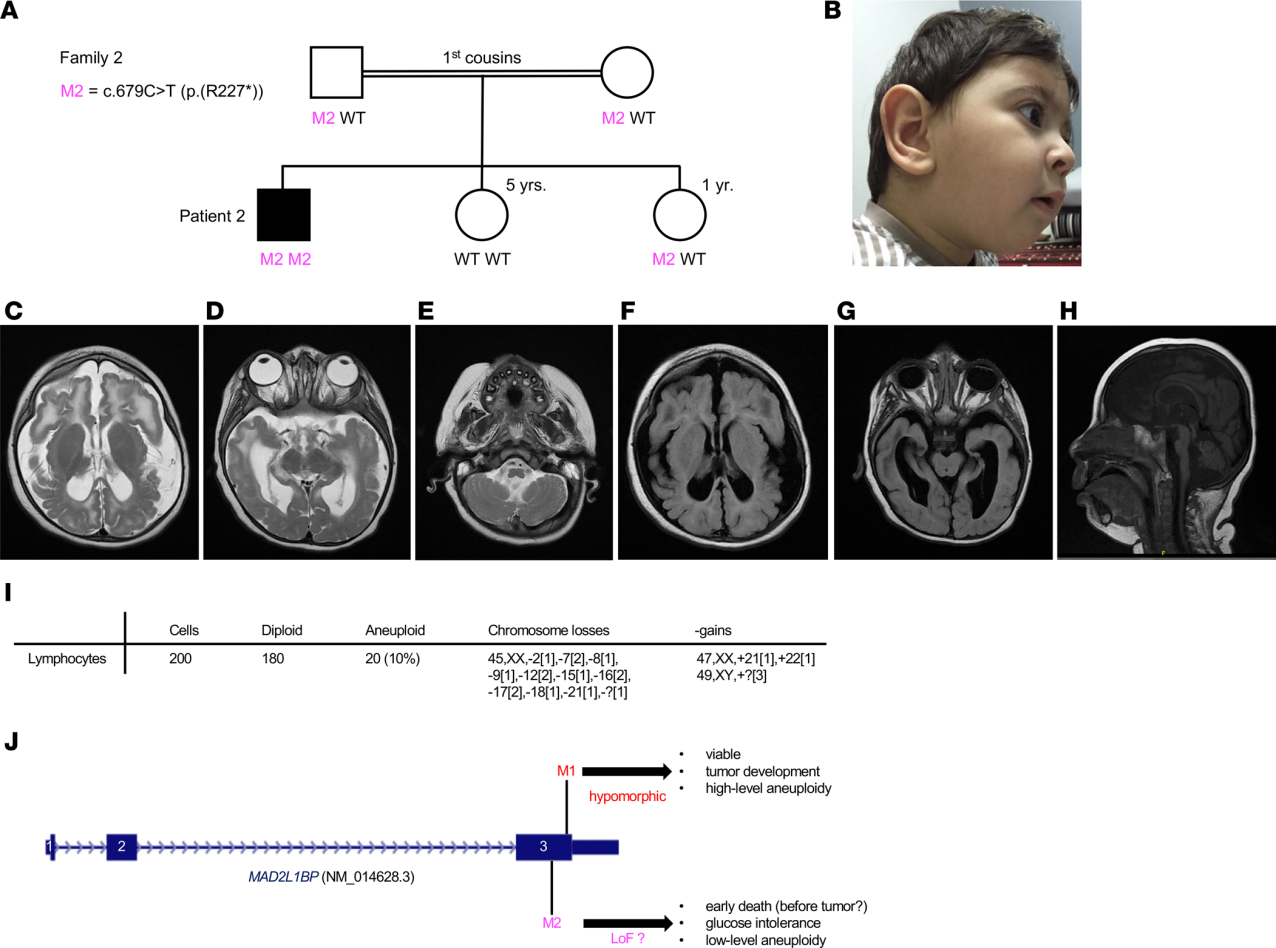
Clinical and genetic findings in patient 2. (**A**) Pedigree. (**B**) Subtle dysmorphism: large ears, microcephaly. (**C**–**H**) cMRI. (**C** and **D**) Diffuse T2 hyperintensity of the white matter and polymicrogyria particularly in the frontal lobes. (**E**–**H**) Generalized brain atrophy, including the cerebellum, the brainstem, and the cervical spinal cord. Thin corpus callosum. (**I**) Results from karyotyping of nontransformed lymphocytes. (**J**) Localization of both homozygous nonsense variants, M1 from patients 1a and 1b and M2 from patient 2, in the *MAD2L1BP* gene and observed disease expression.

Patient 2 (Figure 3) was the first of 3 siblings born to consanguineous parents from Saudi Arabia (first cousins, family 2; Figure 3A). He had microcephaly, severe global developmental delay, refractory seizures, spastic tetraplegia, and major feeding difficulties. cMRI at 3 months showed several abnormalities, including polymicrogyria, a thin corpus callosum, and atrophy of the brain, brainstem, cervical spinal cord, and cerebellum. Blood glucose levels were repetitively found elevated (6–10 mmol/L) in the intensive care unit. While patients 1a and 1b, who, albeit intellectually impaired, showed constant progress in their psychomotor development, patient 2 did not acquire any developmental milestones and died at 23 months of age. No cell cultures were available anymore for functional investigations. For further clinical details of patients from families 1 and 2, see Table 1.

### Homozygous MAD2L1BP nonsense mutations in 2 families with MVA.

In family 1 (Figure 2A), linkage analysis using a reduced panel of 25,000 markers, equally spaced by 100 kb and with a minor allele frequency (MAF) of 0.15, revealed 9 regions of homozygosity by descent (HBD) on chromosomes 3 (54.1 Mb), 6 (3.798 Mb; Supplemental Figure 1; supplemental material available online with this article; https://doi.org/10.1172/jci.insight.170079DS1), 7 (6.32 Mb), 8 (0.658 Mb), 9 (5.16 Mb), 12 (2 regions: 1.76 and 32.82 Mb), 13 (35.83 Mb), and 14 (10.64 Mb), all with the theoretical maximum logarithm of the odds (LOD) score of 1.93 estimated beforehand (Figure 2B). Using 100,000 or all applicable markers of the array did not result in additional HBD regions of significant size (only small regions that were not analyzed further). Whole-exome sequencing (WES) of patients 1a and 1b and their older healthy sister generated 5.9, 10.8, and 17.1 Gb of sequence; 89%, 96% and 99% of the target sequences were covered more than 20 times. Twelve rare (MAF < 1%) homozygous variants were present in both affected siblings, but not in their healthy sister, compatible with the above HBD regions (Supplemental Table 1). Disease causality was considered unlikely if ≥1 of the following applied: a) gene associated with another disease (*RECQL4*, *MED13L*), b) variant annotated homozygously in Genome Aggregation Database (gnomAD) (*ZNF654*, *RECQL4*), c) variant classified as neutral/benign by most prediction programs (8 variants), or d) silent variant not indicative for impaired splicing (*ZNF618*). A rare homozygous nonsense variant, NM_014628.3:c.757C>T [p.(Arg253*), subsequently termed R253*] in *MAD2L1BP* on chromosome 6p21.1 remained (Figure 2, C and D). This variant, rs528509686, has an allele frequency of 3/248,598 in gnomAD (MAF: 0.00001207) with no homozygous individuals annotated (as of November 8, 2022). It resides in the third smallest of the 9 HBD regions (Supplemental Figure 1). This may indicate an origin of the mutation several generations ago. However, since no other individual carrying the same *MAD2L1BP* mutation was found in our sequencing or in huge public and institutional databases, it may have occurred more recently in a small chromosomal segment shared by both parents. Segregation analysis showed heterozygosity in both sisters and the parents (Figure 2A).

In patient 2 (family 2; Figure 3, A–I), chromosomal microarray analysis had revealed a paternally inherited hemizygous deletion of 227 kb on the long arm of chromosome 2 [arr(hg19) 2q21.3(135,214,041-135,441,370)x1 pat], which was considered nonpathogenic. Filtering of the WES data set for *MAD2L1BP* variants now, 8 years after the patient’s death, retrieved the homozygous nonsense variant c.679C>T [p.(Arg227*), subsequently termed R227*]. Segregation analysis showed heterozygosity in the parents and 1 of the 2 healthy sisters, whereas the other sister did not carry the variant (Figure 3A). All of the only 25 *MAD2L1BP* loss-of-function (LoF) variants annotated in gnomAD are extremely rare, and none of them is homozygous. In neither family, there is a history of (aggregated) tumor incidence and thus no indication for an elevated tumor risk for heterozygous carriers.

### Biallelic mutations of MAD2L1BP cause MVA but not an increased mutational rate.

In view of the crucial role of p31^comet^ in mitotic regulation and its close functional and physical relationship to BUBR1 (encoded by *BUB1B*), TRIP13, and BUB1, whose mutations cause constitutional aneuploidy (6–8), extensive karyotyping (G-banding) was carried out for cultured peripheral blood lymphocytes and fibroblasts from skin biopsies of patients 1a and 1b. It revealed high-grade MVA, with singular up to several chromosomes affected per cell (Figure 2, E–H), but not in corresponding chromosome spreads from the older sister, the parents, and the younger sister (prenatal analysis). In patient 2, a normal male karyotype (46,XY) had initially been documented for 20 metaphases. Reanalysis of 200 metaphases from 3 preserved slides archived 8 years ago revealed moderate mosaic aneuploidy, with 10% of cells showing chromosome (mostly) losses and gains (Figure 3I). No *MAD2L1BP* mutations were identified in a heterogeneous cohort of 23 unrelated nonsyndromic patients with sporadic (nonfamilial) occurrence of JGCT (Supplemental Table 2).

Analyzing JGCT DNA of patients 1a and 1b, we identified a comparably large number of somatic mutations with a MAF ≥ 2%. However, closer inspection revealed that only a small fraction of these variants had a MAF ≥ 5%, potentially indicating an increased number of false positives among low-MAF variants (Supplemental Table 3). This assumption was supported by the mutational signature analysis (see below and Supplemental Figures 2 and 3), which indicates that the samples are affected by DNA damage due to being formalin-fixed paraffin-embedded (FFPE). We therefore performed subsequent analysis only for variants with MAF ≥ 5% (although this does not completely eradicate the FFPE damage signature). Considering only these MAF-filtered mutations, the tumor mutational burden (TMB) is estimated to be low in both cases, and microsatellites are predicted to be stable by MANTIS (Supplemental Table 4). Annotation of variants in known oncogenes or tumor suppressor genes revealed no oncogenic alterations in the JGCT of patient 1b. For patient 1a, we found an oncogenic splice region variant in the neurofibromatosis type 1 (*NF1*) gene at very low allele frequency in the tumor tissue (Supplemental Table 5). An additional low-level *NF1* allele deletion was suggested by changes in the relative copy number but not confirmed by the b allele frequencies. It was therefore considered as likely artifact.

### R253* is a hypomorphic mutation that results in partial NMD and residual expression of unstable C-terminally truncated p31^comet^ protein.

**Figure 4:**
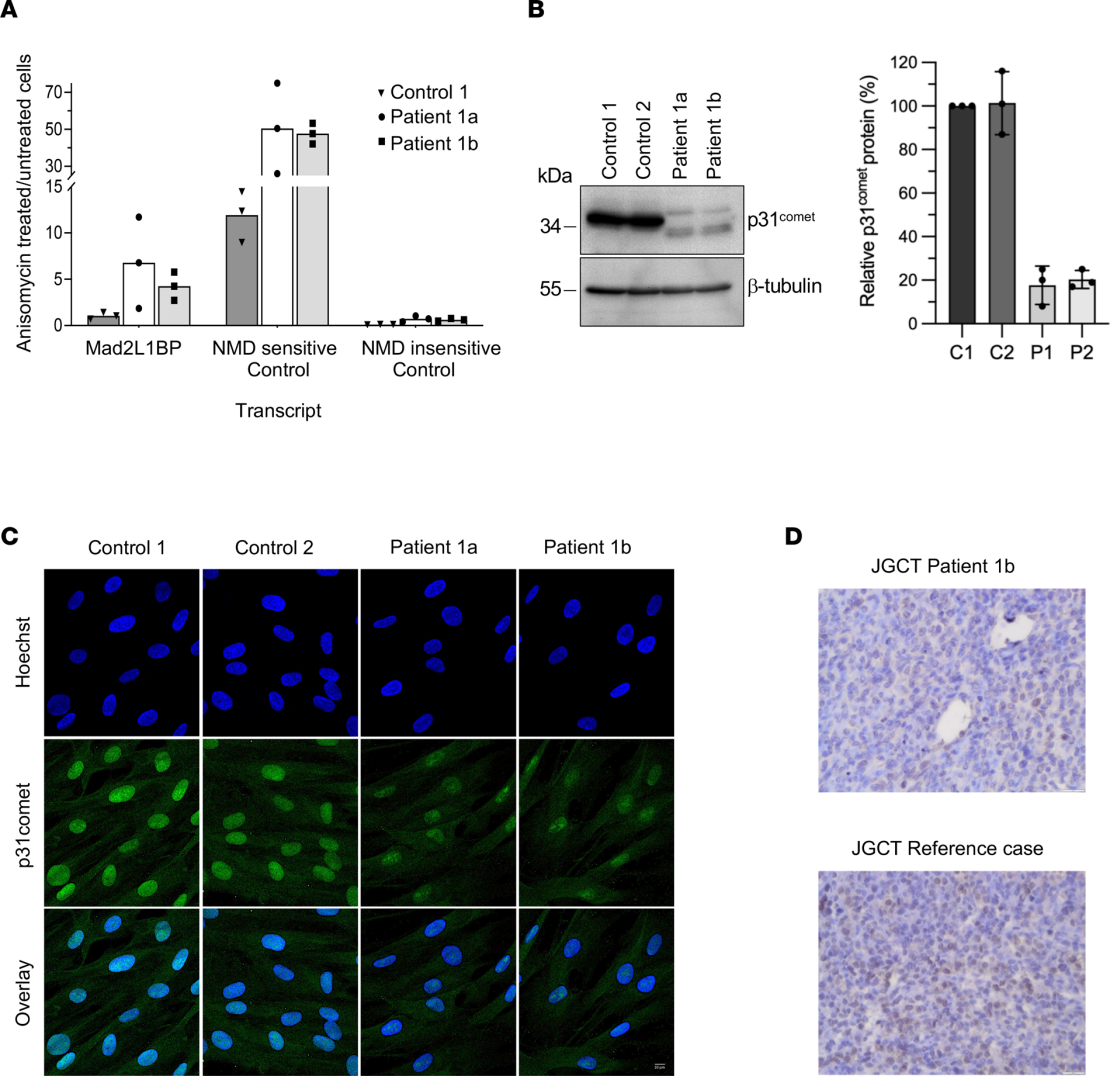
*MAD2L1BP* c.757C>T (R253*) of patients 1a and 1b results in partial NMD and C-terminally truncated p31^comet^ protein. (**A**) Inhibition of NMD in patients’ fibroblasts: increased mRNA expression compared with control cells (ratios of transcript expression in anisomycin-treated/-untreated cells; effect of anisomycin verified by expression change of NMD-sensitive (ENST00000452355.7) compared with NMD-nonsensitive SRSF2 transcript (ENST00000392485.2). Bar plot and scatterplot of 2/3 independent experiments analyzed in duplicates by digital droplet PCR (ddPCR). (**B**) Western blot of fibroblasts showing decreased p31^comet^ protein expression in both patients compared with 2 control samples with p31^comet^ WT. The blot is representative for 3 experiments. Right panel: Intensities of p31^comet^ bands in relation to β-tubulin standard. Quantification was done on 3 Western blots and the lower bands of p31^comet^-R253* were used. All values: means ± SE (error bars). (**C**) p31^comet^ immunofluorescence staining in control and patients’ fibroblasts. Blue, Hoechst staining of DNA; green, p31^comet^. Scale bars, 10 μm. (**D**) p31^comet^ staining of testicular JGCT in patient 1b (above) and in a reference case (below). There is faint nuclear p31^comet^ expression in <5% of tumor cells in patient 1b, compared with approximately 50% in the reference tumor. Original magnification, 40× (in both images); scale bar: 50 μm.

**Figure 5:**
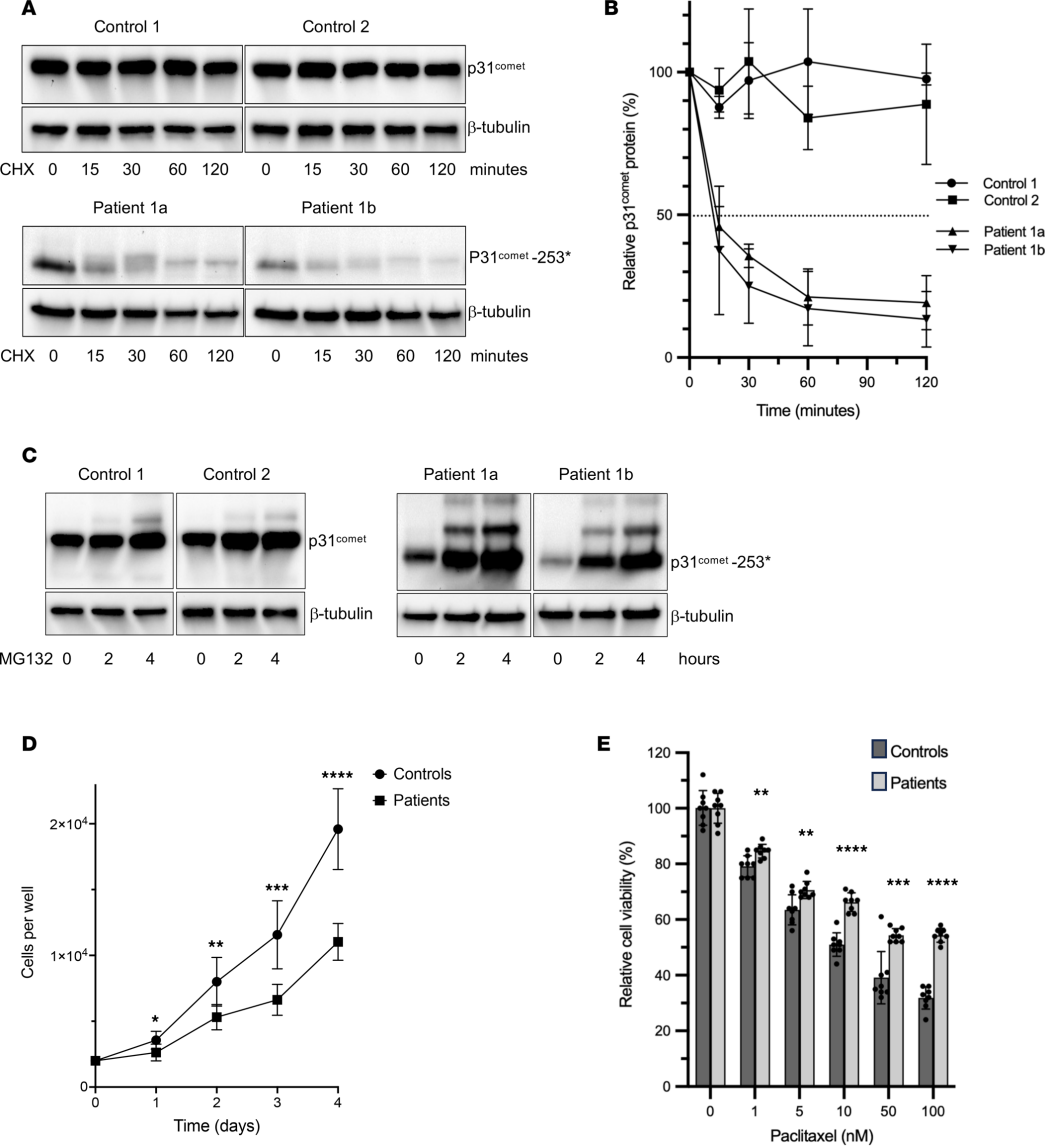
Mutated p31^comet^ is sensitive to proteosomal degradation causing slowed proliferation and decreased sensitivity to chemotherapeutic drugs in patients’ fibroblasts. (**A**) Analysis of p31^comet^ protein half-life using cycloheximide. Fibroblasts were treated with cycloheximide at indicated time points, and p31^comet^ was analyzed by immunoblotting (β-tubulin as internal control). The blots are representative of 3 experiments. (**B**) Densitometric analysis of the immunoblots. Intensities of the p31^comet^ bands in relation to β-tubulin standard. Expression levels were normalized to untreated cells. The dashed line indicates *t_1/2_*. The blots are representative for 3 repeated experiments with consistent results. (**C**) Inhibition of proteasomal degradation by MG132 increases p31^comet^ levels. Fibroblasts were treated with 10 μM MG132 for 2 and 4 hours, and p31^comet^ protein accumulation was analyzed by Western blot (β-actin as internal control). The blots are representative for 3 experiments. (**D**) Growth curves of fibroblasts from 2 controls and both patients. Data were analyzed by a 2-tailed, unpaired Student’s *t* test at each time point. Error bars represent the SD (*n* = 4). (****P < 0.0001; ***P < 0.001; **P < 0.01; *P < 0.05.) Each experiment was repeated 3 times. (**E**) Dose response to paclitaxel. Cell viability of fibroblasts from controls and patients were treated for 72 hours with the indicated concentrations of paclitaxel normalized to DMSO vehicle. Error bars represent the SD (*n* = 4). Data were analyzed by 2-tailed, unpaired Student’s *t* test (****P < 0.0001; ***P < 0.001; **P < 0.01). Each experiment was repeated 3 times.

Chemical inhibition of nonsense-mediated decay (NMD) increased *MAD2L1BP* mRNA levels in fibroblasts from patients 1a and 1b but not in control fibroblasts (Figure 4A). However, residual *MAD2L1BP* mRNA in patients’ fibroblasts not exposed to anisomycin indicated that NMD was incomplete. Indeed, p31^comet^ immunoblots showed a weak band in patients’ fibroblasts that migrated as predicted for p31-R253* (28.3 instead of 31.1 kDa) (Figure 4B). Additionally, a slower migrating protein band was observed, which led us to further analysis of the protein stability (Figure 5, A–C). Compared with control fibroblasts, the level of the truncated protein was reduced by more than 80% (Figure 4B). Using cycloheximide to inhibit translation, we compared the stability of wild-type (WT) p31^comet^ and p31^comet^-R253* by time-resolved immunoblotting. This revealed rapid degradation of p31^comet^-R253* protein, indicating a half-life of approximately 15 minutes (Figure 5, A and B). By contrast, p31^comet^-WT remained stable for at least 2 hours. Levels of p31^comet^-R253* increased upon addition of the 26S proteasome inhibitor MG132 (Figure 5C). This was accompanied by appearance of higher molecular weight (MW) bands, presumably resulting from ubiquitylation of the truncated protein. Thus, the mutation resulted in destabilization of p31^comet^ at both the mRNA and the protein level. Confocal immunofluorescence localization of p31^comet^ in fibroblasts showed that both, the full-length and the C-terminally truncated form, are present mainly in the nucleus and, to a lesser extent, in the cytosol (Figure 4C). Consistent with the Western analyses, p31^comet^-R253* signals appeared weaker, but also more punctate, which might indicate aggregation. In line with residual expression of p31^comet^-R253*, faint staining was observed in JGCT testis tumor tissue of patient 1b (Figure 4D).

### Patient-derived fibroblasts proliferate slower and are less sensitive to chemotherapeutic treatment.

Proliferation assays showed that both patients’ fibroblasts divided more slowly than control fibroblasts (Figure 5D), which might be explained by SAC-mediated mitotic delay (see below) and/or aneuploidy, a hallmark of MVA (7). Fibroblasts were also compared in their sensitivity to the chemotherapeutics paclitaxel (Taxol), which disrupts microtubule dynamics, and etoposide, which inhibits topoisomerase II, thereby giving rise to DNA damage. These analyses revealed that the patients’ fibroblasts were considerably more resistant to paclitaxel and slightly more resistant to etoposide, which is consistent with decreased chemotherapy sensitivity due to aneuploidy (Figure 5E and Supplemental Figure 4) (9).

### Truncated p31^comet^ is unable to support SAC silencing.

**Figure 6:**
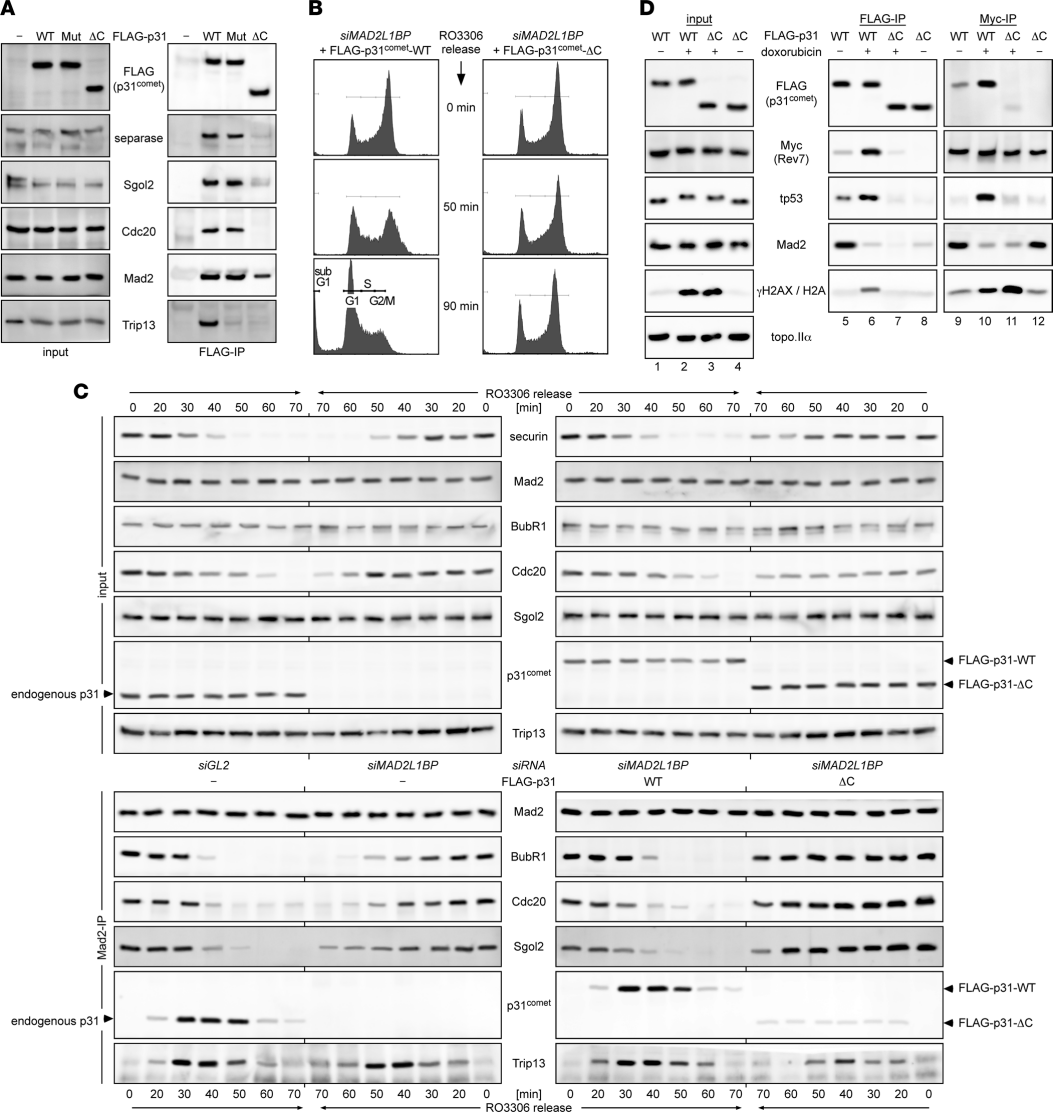
Comparison of p31^comet^-WT and -ΔC in isogenic cell lines. (**A**) C-terminally truncated p31^comet^ cannot associate with Trip13 but retains weak Mad2 binding. Transiently transfected HEK293T cells expressing the indicated FLAG-tagged p31^comet^ variants were Taxol arrested and then subjected to anti-FLAG immunoprecipitation, followed by Western analysis using the indicated antibodies. WT, wild-type (isoform 2, NP_055443.1); PK, Trip13 binding-deficient p31-P228A,K229A; ΔC, amino acids 253–274 deleted. (**B**) Replacing endogenous p31^comet^ by p31^comet^-ΔC greatly delays exit from G_2_/M. HeLaK cells transfected to replace endogenous p31^comet^ by the indicated FLAG_3_-Tev_2_–tagged variants were synchronously released from an RO-336–mediated G_2_ arrest and analyzed by time-resolved immunoblotting (Supplemental Figure 5B) and flow cytometry. (**C**) p31^comet^-ΔC is unable to support Trip13-dependent disassembly of Mad2-containing complexes. HeLaK cells containing the indicated siRNAs were transfected to express FLAG_3_-Tev_2_–tagged p31^comet^-WT or -ΔC or left untreated. Following their release from a G_2_ arrest, cells were subjected to time-resolved immunoblotting either directly (upper panels, “input”) or following Mad2-IP (lower panels). Note that Trip13 association with Mad2 is delayed but still occurs in the absence of p31^comet^; this association is reduced in the presence of p31^comet^-ΔC. (**D**) p31^comet^-ΔC is compromised in Rev7 and tp53 binding. HeLaK cells transiently transfected to express Myc_6_-Rev7 and FLAG_3_-Tev_2_–tagged p31^comet^-WT or -ΔC were treated for 2 hours with doxorubicin (DRB) or carrier solvent (-) and then subjected to IP-Western analyses using the indicated antibodies. Note that the interaction among p31^comet^, Rev7, and tp53 is strongly induced by infliction of DSBs. Note also that Rev7 interacts with Mad2 in undamaged cells, which is consistent with a previous report (65). γH2AX, S139-phosphorylated histone H2AX (marker for DSBs).

We wanted to compare WT and C-terminally truncated p31^comet^ in otherwise isogenic cells and therefore turned from patient-derived fibroblasts to experimentally more tractable cell lines. First, we generated corresponding expression plasmids and confirmed that a truncated ORF encoding amino acids 1 to 252 of p31^comet^ gives rise to an expression product, henceforth designated p31^comet^-ΔC, that runs at the same height in SDS-PAGE as p31^comet^-R253* immunoprecipitated from patients’ fibroblasts (Supplemental Figure 5A). Next, we assessed the ability of p31^comet^-ΔC to interact with Trip13 and Mad2. FLAG-tagged p31^comet^ variants were immunoprecipitated from transfected, Taxol-treated HEK293T cells and copurifying proteins detected by immunoblotting. This IP-Western analysis revealed that p31^comet^-ΔC was unable to interact with Trip13, as might be expected from existing structural data (10), but retained some Mad2-binding activity (Figure 6A). In contrast, recombinant p31^comet^-WT (in its 274-residue isoform 2) interacted with Trip13 and Mad2 and (via Mad2) with Cdc20, Sgo2, and separase, as reported (4, 5, 11). Given that Trip13 both activates and inactivates SAC signaling (12), the functional consequences of p31^comet^-ΔC for mitosis were hard to predict. To clarify this issue, endogenous p31^comet^ was depleted from HeLaK cells and replaced by transfection of siRNA-resistant expression plasmids encoding FLAG-tagged p31^comet^-WT or -ΔC. Following synchronization in G_2_ phase, cells were released and analyzed by flow cytometry and time-resolved immunoblotting. While most p31^comet^-WT-expressing cells had entered G_1_ phase 90 minutes after release, most p31^comet^-ΔC-expressing cells were still in G_2_/M phase at this time (Figure 6B). This was accompanied by greatly delayed kinetics of separase activation (as judged by autocleavage) and securin and cyclin B1 degradation, as well as Cdc27 dephosphorylation (Supplemental Figure 5B). The delay of late mitotic events in cells that relied on p31^comet^-ΔC was verified when the above experiment was repeated with prometaphase-arrested cells that were released by chemical inhibition of the SAC-kinase aurora B (Supplemental Figure 6A). Thus, replacing endogenous p31^comet^ by p31^comet^-ΔC greatly prolongs metaphase. Additional Mad2-IPs revealed that the reason for this delay is the inability to disassemble the MCC (Mad2-Cdc20-BubR1-Bub3) and the Mad2-Sgo2-separase complex (Figure 6C and Supplemental Figure 6B). At first sight, the inability of p31^comet^-ΔC to support SAC inactivation (Figure 6, B and C; Supplemental Figure 5B; and Supplemental Figure 6) seems to be at odds with the decreased sensitivity of the patients’ fibroblasts to paclitaxel (Figure 5E). This is because this spindle toxin hyperactivates the SAC and, hence, is expected to act synergistically with p31^comet^ inactivation, resulting in permanent mitotic arrest and, ultimately, cell death (13). However, while some paclitaxel-treated cell lines die in mitosis, most only die in G_1_ phase following mitotic slippage (14, 15). If the patients’ fibroblasts belonged to this latter group, a more robust arrest might actually protect them from apoptosis. An alternative explanation might just lie in the slow proliferation of the patient-derived fibroblasts (Figure 5D), which has been reported to increase resistance of aneuploid cells against chemotherapeutics (9) and could also explain the slightly increased resistance of the patients’ cells against etoposide (Supplemental Figure 4). Interestingly, late mitotic events were delayed but eventually occurred in cells that were just depleted of p31^comet^ and did not express p31^comet^-ΔC (Figure 6C). This correlated with transient, p31^comet^-independent association of Trip13 with Mad2, which was partially suppressed in the presence of p31^comet^-ΔC (Figure 6C). We speculate that this phenotype is the result of residual Mad2 binding upon overexpression of the truncated p31^comet^ and does not occur under the low in vivo concentrations of p31^comet^-R253* (see Discussion).

### Truncation of p31^comet^ shifts DSB repair from HDR to NHEJ.

Also, p31^comet^ protein mediates the Trip13-dependent disassembly of the Rev7-containing shieldin complex, thereby promoting HDR of double-strand breaks (DSBs) and poly ADP ribose polymerase inhibitor resistance (16, 17). To clarify whether p31^comet^-ΔC retained this function, we first conducted reciprocal co-IP experiments from transfected HeLaK cells expressing Myc-tagged Rev7 and FLAG-tagged p31^comet^-WT or -ΔC. As expected, p31^comet^-WT interacted with Rev7, and this interaction was strongly enhanced by prior cell treatment with the radiation mimetic doxorubicin (Figure 6D, lanes 5, 6, 9, 10). In contrast, p31^comet^-ΔC exhibited minimal Rev7 binding that became detectable only upon infliction of DSBs (lanes 7, 8, 11, 12). This correlated with enhanced association of Rev7 with chromatin (as judged by co-IP of histone H2A) in p31^comet^-ΔC– over p31^comet^-WT–expressing cells, indicative of p31^comet^-ΔC’s inability to aid in Trip13-dependent extraction of Rev7 from DNA (lanes 9–12). To assay DSB repair, we capitalized on 2 U2OS reporter lines, in which the repair of an induced, single DSB results in GFP expression only if it occurs by HDR in the one cell line and by NHEJ in the other (18, 19). Detection of GFP by Western blot and quantification of GFP-positive cells by flow cytometry revealed that RNAi-mediated depletion of endogenous p31^comet^ compromised HDR to a similar extent as did knockdown of separase (Supplemental Figure 7, lanes 1, 4, 5) (20). Importantly, HDR in p31^comet^-depleted cells was rescued upon expression of siRNA-resistant, transgenic p31^comet^-WT but not p31^comet^-ΔC (lanes 5–7). Moreover, p31^comet^-WT but not p31^comet^-ΔC inhibited NHEJ upon overexpression, albeit not as much as chemical inhibition of ligase IV with SCR7 (lanes 8–10, 12, 14). Taken together, these results show that the association of p31^comet^ with Trip13 is fully abolished in the ΔC variant, while its binding to Mad2 and Rev7 is greatly compromised. Consequently, the known p31^comet^ functions of SAC inactivation, separase activation, and HDR promotion are crippled in p31^comet^-ΔC, at least when endogenous p31^comet^ is acutely replaced by this truncated variant, leaving cells no time to activate any compensatory mechanisms.

### p31^comet^ and Rev7 interact with the tumor suppressor tp53.

Motivated by a reported 2-hybrid interaction of p31^comet^ with tp53 (21), we asked whether p31^comet^ would also associate with tp53 in human cells. Therefore, the FLAG-IPs from HeLaK cells expressing FLAG-tagged p31^comet^-WT or -ΔC and MYC-tagged Rev7 (see above) were also analyzed by immunoblotting for endogenous tp53. Of note, this did reveal copurification of tp53 with p31^comet^-WT but not -ΔC (Figure 6D, lanes 5, 8). The tp53-p31^comet^-WT interaction was enhanced upon infliction of DSBs (lanes 5, 6), and this was not due to DNA damage–induced tp53 stabilization because it occurred prior to increase of tp53 levels (lanes 1–4). Interestingly, in DRB-treated cells, tp53 also copurified with MYC-tagged Rev7 (lane 10). We cannot exclude that these interactions are indirect and bridged by chromatin/DNA, but we consider this unlikely for the following 2 reasons: 1) Cell lysates were extensively nuclease-treated to maximally fragment DNA prior to IP. 2) The binding of Rev7 and p31^comet^ to tp53 does not always correlate with their binding to histone H2A/chromatin. For example, Rev7’s binding to tp53 was lost upon expression of p31^comet^-ΔC, while its association with H2A simultaneously increased (lanes 10,11). Furthermore, p31^comet^ interacted with tp53 but not H2A in undamaged cells (lane 5). We therefore propose that p31^comet^-WT and Rev7 are binding partners of tp53. Moreover, binding is induced by DNA damage and abrogated by C-terminal truncation of p31^comet^.

## Discussion

MVA is an ultrarare (worldwide prevalence of <1/1,000,000, according to Orphanet) autosomal recessively inherited condition characterized by constitutional mosaicism for aneuploidy (chromosome gains and losses of variable proportion, but usually affecting ≥25% of cells) and variable expression of prenatally manifesting growth retardation, microcephaly, developmental delay, childhood tumors, eye anomalies, and mild dysmorphism (22–24). Of the 8 genes currently associated with MVA or, in the case of *BUB1*, *MAD1L1*, *SLF2*, and *SMC5*, closely related conditions (6–8, 25–28), 5 (*BUB1B*, *TRIP13*, *CEP57*, *BUB1*, and *MAD1L1*) encode proteins of the mitotic checkpoint machinery and the centrosome. Tumor predisposition has been observed in patients with biallelic LoF of *BUB1B*, *TRIP13*, and *MAD1L1*, with resulting elevated risks for rhabdomyosarcoma, Wilms tumor, and a variety of malignancies, respectively (7, 8, 27). The recent identification of heterozygous germline *CDC20* missense variants cosegregating with ovarian germ cell tumors and resulting in impaired binding to BUBR1 and mitotic slippage further demonstrates that proteins associated with MVA or, more generally, the SAC and APC/C-regulatory network, represent candidates for monogenic tumor entities (29).

We report on 3 patients from 2 families with homozygous *MAD2L1BP* nonsense variants and microcephaly, epileptic encephalopathy with brain malformations, and impaired growth and psychomotor development. Both affected siblings in family 1, patients 1a and 1b, developed JGCTs. The tumor predisposition in largely p31^comet^-deficient individuals is noteworthy because overexpression of 1 substrate of p31^comet^-mediated inhibition, MAD2, in mice initiates the formation of different neoplasias (mostly malign, such as carcinomas, lymphomas, but also adenomas), likely through the observed chromosomal instability (CIN) with whole-chromosome gains and losses (30).

The occurrence of JGCT in siblings of both sexes (affecting the respective target organs, ovary and testis) is striking and indicates that biallelic p31^comet^ germline mutations predispose for this (especially in infancy) extremely rare and usually sporadic gonadal tumor type. JGCTs may occur in enchondromatosis-associated Ollier disease and Maffucci syndrome, both representing likely nonhereditary conditions, in which somatic *IDH1* and *IDH2* mutations have been reported (31). There are only a few descriptions in the context of germline mutations in patients, e.g., a 12-year-old girl with ovarian JGCT and homozygosity for a pathogenic biallelic variant of *MBD4* (32), encoding a glycosylase involved in repair of G:T mismatches; (after the occurrence of other tumors) ovarian JGCT at 16 months of age in a girl with simultaneous heterozygous pathogenic variants of *PTEN* (splice site) and *TP53* (missense) (33); and in a 23-year-old woman with a heterozygous *TP53* nonsense variant (34).

In this context, it is noteworthy that we found p31^comet^ and tp53 to interact and that the p31^comet^-R253* mutation abrogates this interaction (Figure 6D). In addition, we find that tp53 interacts with Rev7 (Figure 6D). Depletion of endogenous p31^comet^ from HEK293T cells results in increased Rev7-tp53 association (SH and OS, unpublished observation), which suggests that the Rev7-tp53 interaction is direct. Furthermore, based on what is known about other Rev7 complexes (35), it is conceivable that WT p31^comet^ will act as an adaptor for the Trip13-dependent disassembly of the Rev7-tp53 complex. We currently do not know whether Rev7 regulates transcriptional activity of tp53, whether tp53 impacts Rev7’s functions in DNA damage repair, or whether the regulation is mutual. Hence, it is unclear if the dysregulation of the Rev7-tp53 interaction might contribute to the syndrome reported here. However, loss of tp53 function is known to result in CIN (36–38), and one can speculate that loss of a tp53 binding partner contributes to JGCT formation in patients 1a and 1b.

p31^comet^, as an antagonist of Rev7, impacts the repair of DSBs and replication-stalling DNA lesions (35), but we found no indication for an increased mutational burden. However, through stabilization of both shieldin and polymerase ζ, loss of p31^comet^ function will merely result in preferential repair of DSBs by NHEJ rather than HDR and a putative increase in translesion synthesis activity, respectively, and both these consequences would not necessarily result in an obvious increase of the mutational rate. Hence, the tumors in patient 1a and 1b may largely result from their high-grade aneuploidy — considering the lack of tumors in other MVA subtypes, possibly in combination with other genetic factors (e.g., the impaired interaction with tp53; see above).

As a negative regulator of Mad2 and Rev7, p31^comet^ promotes anaphase onset and DNA repair by homologous recombination and translesion synthesis (Supplemental Figure 8) (35). In all cases studied so far, p31^comet^ fulfills its functions by acting as an adaptor that recruits the AAA-ATPase Trip13 to HORMA protein–containing complexes, thereby facilitating their active disassembly. A murine knockout model showed that insulin signaling is an additional important function of p31^comet^. Cells from these mice display slowed proliferation, with elevated apoptosis, decreased S phase, increased G_2_/M population, and low-level aneuploidy (39). Of note, and unlike patients 1a and 1b, patient 2 presented with only low-level aneuploidy (10%), but repeated episodes of severe blood glucose elevations and early death (23 months), all key findings in p31^comet–/–^ mice. The p31^comet^-R227* mutation in patient 2 predicts truncation of an additional 26 residues compared with patients 1a and 1b, possibly further reducing p31^comet^ function compared with R253*-associated p31^comet^ protein. Patient 2 and p31^comet–/–^ mice may lack (detectable) tumor development due to their early death but also due to only minor aneuploidy (Figure 3J). Early lethality of p31^comet–/–^ mice was attributed to impaired insulin signaling and decreased hepatic glycogen stores. In the case of patient 2, who died 8 years ago and had severe epileptic encephalopathy, recurrent hyperglycemia episodes had been documented when he was in the neonatal intensive care unit. It is unclear, though, if the latter reflected constitutively defective insulin signaling, and if it did, if this was due to the biallelic *MAD2L1BP* nonsense variant. Since phenotype severity may apparently vary significantly even between siblings (patient 1b has less pronounced brain malformations than his sister and no psychomotor impairment), phenotypes may be even more variable between unrelated carriers of biallelic pathogenic *MAD2L1BP* variants. In this regard, the dissimilar disease expression in carriers of biallelic pathogenic variants in another MVA gene, *BUB1B*, is mentionable: the initially reported 5 families with the *BUB1B*-associated MVA1 subtype displayed an — albeit variable — childhood-onset MVA phenotype with intrauterine growth retardation, microcephaly, neurological abnormalities, and, in 2 families, cancer (7). In contrast, another patient was healthy until he developed multiple gastrointestinal adenocarcinomas, at the age of 34 years and 2 decades later; mosaic aneuploidy in his cells, but no MVA1-related clinical abnormality was detected subsequent to the identification of a homozygous pathogenic *BUB1B* variant (40).

Because we did not have enough patient cells, our co-IP experiments had to be carried out in vitro. It cannot be excluded that protein interactions may partially differ from the in vivo situation. Our investigations revealed that p31^comet^-ΔC is unable to support SAC inactivation or to promote HDR of DSBs (Figure 6, B and C; Supplemental Figure 5B; and Supplemental Figures 6 and 7). Moreover, while depletion of endogenous p31^comet^ delays late mitotic events in HeLaK cells, overexpression of siRNA-resistant p31^comet^-ΔC, rather than rescuing this phenotype, further aggravates it (Figure 6C). Thus, p31^comet^-ΔC seems to have an inhibitory effect on MCC disassembly. This unexpected observation raises several questions: If cells are better off without p31^comet^ than with p31^comet^-ΔC, why do knockout mice die early (as did patient 2, in contrast with patients 1a and 1b)? Why are fibroblasts of patients 1a and 1b, which solely express residual p31^comet^-R253*, still able to proliferate (albeit at a reduced rate) and do not terminally arrest in mitosis? How can the impact of p31^comet^-ΔC in vitro be reconciled with the fact that the corresponding *MAD2L1BP* c.757C>T allele in vivo is recessive? Apparently, p31^comet^-R253* is sufficient to maintain vital insulin signaling in patients 1a and 1b. We further speculate that p31^comet^-R253*, which is present only at low level due to destabilization of both protein (likely mediated by ubiquitin-mediated proteolysis) and mRNA (Figure 4, A and B, and Figure 5, A–C), prevents constitutive SAC-mediated arrest in mitosis. How can p31^comet^-ΔC still function in insulin signaling but inhibit SAC inactivation, if both processes depend on binding of p31^comet^ to Mad2? Our pull-down experiments revealed that p31^comet^-ΔC retained weak Mad2 binding but was fully defective in Trip13 interaction (Figure 6A). Thus, by binding to Mad2, this truncated p31^comet^ variant might shield the MCC from Trip13-dependent disassembly. Consistently, the Mad2-Trip13 interaction, which was delayed but still occurred in the absence of p31^comet^, was compromised by p31^comet^-ΔC (Figure 6C). In contrast, binding of p31^comet^-R253* to insulin receptor–associated (IR-associated) Mad2 may suffice to prevent IR endocytosis (39) and, hence, sustain insulin signaling even in the absence of Trip13-dependent disassembly.

## Methods

### Patients

Blood samples were collected from family members (parents, patients, and their healthy siblings), and DNA extraction was performed using standard procedures. Family 1 is of Egyptian and family 2 of Saudi Arabian origin. The parents are consanguineous (first cousins) in both families.

### Genetic analyses

#### Family 1.

Extensive karyotyping was carried out for all family members except the youngest daughter from cultured peripheral lymphocytes (and for both patients, also from cultured fibroblasts derived from a skin biopsy) following standard procedures. For the youngest daughter, extensive prenatal chromosome analysis was performed after chorionic villi sampling. Genome-wide linkage analysis with samples from both affected siblings, the older unaffected sister, and their parents was carried out using the Axiom Precision Medicine Research Array (Thermo Fisher Scientific). Genotypes were called by the Axiom Analysis Suite v4.0 or v5.0, respectively. Subsequent data handling was performed using the graphical user interface ALOHOMORA (41). Relationship errors were identified by the program Graphical Relationship Representation (42). The program PedCheck was applied to find Mendelian errors (43), and data for SNPs with such errors were removed from the data set. Non-Mendelian errors were identified by using the program MERLIN (44), and unlikely genotypes for related samples were deleted. Linkage analysis was performed assuming autosomal-recessive inheritance, full penetrance, consanguinity, and a disease allele frequency of 0.0001. Multipoint LOD scores were calculated using MERLIN. Haplotypes were reconstructed with MERLIN and presented graphically with HaploPainter (45). WES was carried out with DNA from both patients and their older healthy sister. Library preparation was performed using the HyperPlus kit (KAPA Biosystems, Roche) followed by enrichment with xGen Exome Research Panel v1.0 (IDT Integrated Technologies). In short, 100 ng of genomic DNA was fragmented to a peak size of 150–200 bp using KAPA Frag enzyme. The fragmented genomic DNA was end-repaired, A-tailed, ligated to single-indexed adapters, and PCR-amplified according to manufacturer’s instructions. PCR reactions were purified using KAPA Pure Beads. For target capture, 500 ng of pooled libraries (8 libraries per pool) were hybridized to biotinylated oligonucleotides at 65°C for 4 hours. The captured libraries were pulled down using Dynabeads M-270 Streptavidin (Life Technologies, Thermo Fisher Scientific). A postcapture PCR was carried out to amplify the captured libraries and add the barcode sequences for multiplex sequencing for 12 cycles. Subsequently, amplified libraries were cleaned with Agencourt AMPure XP Beads (Beckman Coulter). Each captured library was quantified and validated using a Qubit fluorometer (Thermo Fisher Scientific) and Bioanalyzer high-sensitivity chips (Agilent Technologies). The pooled libraries were paired-end sequenced on an Illumina NextSeq 500 system. Read mapping against the hg19 human reference genome, variant calling, annotating, and filtering against 1000 Genomes and ExAC were performed with the SeqNext module of SeqPilot software (JSI Medical Systems). The cutoff for the maximum MAF was set to 1%. Searches for disease-relevant variants in genes associated with the Human Phenotype Ontology terms “Microcephaly” (HP:0000252), “Short stature” (HP:0004322), and “Global developmental delay” (HP:0001263) and for pathogenic homozygous or potentially compound-heterozygous variants were performed with the software varSEAK Pilot 2.0.2 (JSI Medical Systems). Variants were checked for their presence in the disease-specific variant databases ClinVar and HGMD. Nonsense, frameshift, and canonical splice site variants were regarded likely pathogenic. Small nucleotide variants (SNVs) were assessed using scores from the pathogenicity prediction algorithms DANN, MutationTaster2, FATHMM, FATHMM-MKL, MetaSVM, MetaLR, LRT, MutationAssessor, SIFT, and PROVEAN as well as conservation scores from GERP++, phyloP20way, phyloP100way, SiPhy29way, fitCons-gm, phastCons20way, and phastCons100way compiled by the dbNSFP database (46). In addition, the CCG pipeline and interface (Varbank 2.0; https://varbank.ccg.uni-koeln.de/varbank2/) were used for data analysis as described previously (47, 48). ExAC and gnomAD databases (49) (as of February 2021) were searched for the homozygous candidate variants. PCR amplification of *MAD2L1BP* exon 3 and subsequent Sanger sequencing were carried out for segregation analysis in the reported family.

#### Family 2.

Patient 2 had been subjected to karyotyping of blood lymphocytes and WES as described previously (50) (Department of Human Genetics, Radboud University Medical Centre, Nijmegen, Netherlands) and chromosomal microarray analysis (Affymetrix CytoScan HD; Mayo Clinic) before he died 8 years ago. Segregation analysis was carried out as in family 1 in the sisters and the parents.

#### Mutation screening in patients with sporadic JGCT.

WES data from FFPE tumor tissues or adjacent normal tissues of 23 unrelated patients with JGCT (1 male with testicular JGCT, 22 females with ovarian JGCT, age at diagnosis ranging from 1 month to 22 years) were screened for *MAD2L1BP* mutations. Because somatic hotspot mutations of *FOXL2* [c.402C>G; p.(Cys134Trp)] are relatively common in adult GCTs (51), analyses included this variant, too. Furthermore, since somatic pathogenic *DICER1* variants have been reported in JGCT (52), WES data were also analyzed for variants in this gene. For none of those patients, familial occurrence of JGCT was reported.

### Skin fibroblast isolation and culture

No cells were available anymore from patient 2, who died 8 years ago. In the case of patients 1a and 1b, fibroblasts were isolated from dermal punch biopsies of the upper arm as previously described (53). In short, skin biopsies were cut in 18–24 equally sized pieces containing all skin layers. The pieces were plated on a 6-well plate that was coated with 0.1% gelatin. Fresh media were added every other day. Fibroblasts migrated out of the skin biopsies after 7–10 days and were split on two 75 cm^2^ flasks after 3–4 weeks. When reaching 90% confluence, the flasks were split on three 175 cm^2^ flasks. From here, fibroblasts were expanded or frozen in liquid nitrogen for long-term storage. Fibroblasts were routinely cultured in IMDM/Glutamax containing 15% fetal bovine serum and 1% penicillin/streptomycin (all Invitrogen, Thermo Fisher Scientific).

### Cell proliferation analysis

Fibroblasts of patients and healthy donors were seeded in quadruplicates in 24-well plates at 2,000 cells/well. Cells were counted at the indicated days and a growth curve was calculated. Error bars represent standard deviation of the mean. Statistical significance of the patients’ and control fibroblasts was evaluated each day by an unpaired, 2-tailed Student’s *t* test (*P* < 0.05 considered statistically significant).

### Cell viability assay

Fibroblasts of patients and healthy donors were seeded in quadruplicates in 96-well plates at 500 cells/well and allowed to adhere overnight. Medium was then removed and cells were provided with medium containing etoposide (Selleckchem, S1225) or paclitaxel (Selleckchem, S1150) at the indicated concentrations. DMSO was added as vehicle control. After 72 hours, cell viability was determined using CellTiter-Glo Luminescent Cell Viability Assay (Promega), according to the manufacturer’s instructions. Luminescence was measured in a microplate plate reader (Tecan). Percentage cell growth was calculated relative to the corresponding DMSO-treated cells, and error bars indicate standard deviation of the mean (*n* = 4). Statistical significance of the patients’ and control fibroblasts was evaluated on the highest dose by an unpaired, 2-tailed Student’s *t* test (*P* < 0.05 considered statistically significant).

### Assessment of NMD

Cultured fibroblasts (80%–90% confluence) of both patients and a control proband were incubated with anisomycin (1 μg/mL) for 6 hours. RNA isolation and reverse transcription were performed as described previously (54). The expression changes of *MAD2L1BP* and the NMD control transcript SRSF2 after anisomycin treatment were analyzed by ddPCR (QX200 Droplet Digital PCR System, Bio-Rad Laboratories).

### Assay for protein stability and degradation and Western blot experiments

For protein half-life experiments, fibroblasts were treated with 100 μg/mL cycloheximide (Selleckchem) or DMSO as control. To examine whether the truncated p31^comet^ protein was degraded by ubiquitin-proteasome pathway, 10 μM MG132 (Selleckchem) was used to inhibit proteasome-dependent protein degradation. Cells were taken at the indicated time points. Western blots were performed using antibodies against p31^comet^ (Invitrogen, Thermo Fisher Scientific; PA5-65029, 1:1,000 dilution). Equal loading of protein was demonstrated by probing the membranes with anti–β-tubulin (MilliporeSigma SAB4200732, 1:5,000 dilution).

### Immunofluorescence staining

Fibroblasts were seeded in 8-well chamber slides (Falcon, Corning), allowed to adhere overnight, and fixed with 4% paraformaldehyde. Immunostaining was performed using antibody against p31^comet^ (1:200) and Alexa Fluor 488–labeled anti-rabbit antibody as secondary antibodies (Invitrogen, Thermo Fisher Scientific; catalog A-11034, 1:400). Counterstaining of nuclei was performed with Hoechst 33342 (Invitrogen, Thermo Fisher Scientific). The cells were imaged directly in the chambers using a Zeiss LSM 710 NLO confocal laser scanning microscope, and image analysis was performed using the Zeiss Zen-2009 software (Carl Zeiss Microscopy).

### FFPE staining

Immunohistochemical stains were performed on ultrathin sections of FFPE material. Deparaffinization, target retrieval, immunohistochemical staining, and counterstaining were performed onboard the fully automated EnVision FLEX High pH (Agilent Technologies) according to manufacturer’s staining protocol for estrogen receptor α (monoclonal rabbit anti-human, clone EP1, IR 084, Agilent Technologies) and for progesterone receptor (monoclonal mouse anti-human, clone PgR 1294, GA090, Agilent Technologies). Both primary antibodies were used for immunohistochemical epitope staining for 10 minutes after heat-induced epitope retrieval using diluted EnV FLEX TRS High pH (50×) at 97°C for 30 minutes. Epitope visualization was done by EnV FLEX Substrate Working Solution (Dako) resulting in brown nuclear signal. For inhibin (monoclonal mouse anti-human, clone R1, GA058, Agilent Technologies), staining was performed using EnV FLEX High pH for target retrieval 20 minutes at 97°C and antibody incubation for 25 minutes. Visualization was done by EnV FLEX Substrate Working Solution in combination with EnVision FLEX+Mouse LINKER (GV821, Agilent Technologies). Cellular staining pattern is cytoplasmatic. For human p31^comet^ staining of testicular JGCT from patient 1b and a reference testicular JGCT, we used the unconjugated mouse MAD2L1BP antibody OTI3C11 (Thermo Fisher Scientific). The staining program was performed manually. The antibody was diluted 1:100 with an incubation time of 30 minutes and high pH. Nuclear counterstain was done using hematoxylin solution. FFPE material was also routinely stained for H+E, PAS and reticulin. Stained slides were examined by light microscope Olympus BX53 and photographed with Axiocam 208 color (Zeiss).

### Antibodies and immunoprecipitation

The following antibodies used for immunoblotting were previously described: rabbit anti-separase (55), mouse anti-GFP (20), rabbit anti-p31^comet^, rabbit anti-Sgo2 (anti-DVPPRESHSHSDQSSKC), and guinea pig anti-TRIP13 (11). Other antibodies included mouse anti-FLAG (1:2,000; MilliporeSigma, M2), mouse anti-securin (1:1,000; MBL, clone DCS-280), mouse anti–cyclin B1 (1:1,000; MilliporeSigma 05-373), rabbit anti-Mad2 (1:1,000; Bethyl A300-300A), mouse anti-Mad2 (1:1,000, Santa Cruz Biotechnology, clone 17D10), rabbit anti-p53 (1:2,000; Abcam ab131442), rabbit anti-H2A (1:500; Abeomics 11-7017), rabbit anti–phospho Ser139-histone H2A.X (γH2AX; 1:5,000; Merck 6L16), mouse anti-Cdc27 (1:1,000; MilliporeSigma, clone AF3.1), mouse anti–topoisomerase IIα (1:1,000; Enzo Life Sciences, clone 1C5), rat anti-HA (1:2,000; Roche, clone 3F10), mouse anti-BubR1 (1:1,000; BD Biosciences, clone 9/BUBR1), mouse anti-Cdc20 (1:800; Santa Cruz Biotechnology, clone H7), and mouse anti–α-tubulin (hybridoma supernatant 1:200; DSHB, 12G10). Secondary antibodies for immunoblotting were horseradish peroxidase–conjugated goat anti-rabbit, anti-mouse, anti-rat, and anti-guinea pig IgGs (1:20,000, 12-348, 12-349, AP202P, AP108P, respectively; MilliporeSigma). For immunoprecipitation experiments, the following affinity matrices and antibodies were used: mouse anti-FLAG Agarose (MilliporeSigma, M2), mouse anti–c-Myc Agarose (MilliporeSigma, A7470), rabbit anti-Mad2 (Bethyl, A300-300A), rabbit anti-p31^comet^ (see above), and unspecific rabbit control IgGs (12-370 MilliporeSigma). For noncovalent coupling of rabbit IgGs, 4 μg antibody was rotated with 10 μL of packed protein A-sepharose (GE Healthcare, now Cytiva) in the presence of 1% w/v BSA (Roth) for 90 minutes at room temperature and then washed 3 times to remove unbound antibody. Cell pellets were lysed with a dounce homogenizer in fresh lysis buffer (20 mM Tris-HCl pH 7.7, 100 mM NaCl, 10 mM NaF, 20 mM β-glycerophosphate, 15 mM MgCl_2_, 0.1% Triton X-100, 5% glycerol), combined with benzonase (30 U/L), and incubated on ice for 1 hour. To preserve protein phosphorylation status, lysis buffer was additionally supplemented with 100 nM calyculin A (LC Laboratories). Crude lysates were centrifuged at 4°C and 2,500*g* for 5 minutes to give a low-speed supernatant, which still contained fragmented chromatin, or at 4°C and 16,000*g* for 30 minutes to remove chromatin. Subsequently, 10 μL of antibody-carrying beads were incubated with 1 mL cell lysate for 4 hours or overnight at 4°C and washed 5 times with lysis buffer before bound proteins were eluted by boiling in SDS sample buffer.

### Cell lines and treatments

HeLaK and HEK293T cells were gifts from Dirk Gerlich (Vienna, Austria) and Marc Kirschner (Boston, Massachusetts, USA), respectively. The DR-GFP and EJ5-GFP reporter U2OS cell lines were provided by Maria Jasin (New York, New York, USA) and Jeremy Stark (Duarte, California, USA), respectively. All these cell lines were cultured in DMEM (Biowest) supplemented with 10% FCS (MilliporeSigma) at 37°C and 5% CO_2_. To ensure contamination-free conditions, cells were regularly treated with 1× MycoXpert (Capricorn). pCS2-based plasmids were used for ectopic expression of the following proteins: N-FLAG_3_-Tev_2_-p31^comet^-WT (hCMT2, isoform2, NP_055443), N-FLAG_3_-Tev_2_-p31^comet^-ΔC (amino acids 1 to 252, truncated protein corresponding to the *MAD2L1BP* mutation of patients 1a and 1b) or N-FLAG_3_-Tev_2_-p31^comet^-P228A, K229A (Trip13-binding deficient), N-Myc_6_Tev_2_-hMad2L2 (Rev7), and HA-ER-I-SceI (estrogen receptor–tagged homing endonuclease) (20). HEK293T cells were transfected using a calcium phosphate–based method. HeLaK or U2OS cells were transfected with Lipofectamine 2000 or PEI (VWR, linear, MW 25,000) in the case of plasmids and with RNAiMax (Invitrogen, Thermo Fisher Scientific) in the case of siRNA duplexes (Dicer-substrate siRNAs [DsiRNAs] from IDT). For knockdown of separase (encoded by *ESPL1*) a single siRNA duplex (sense: 5′-AACUGUUCUACCUCCAAGGUUAGAUUU-3′) was used at a final concentration of 70 nM. In the case of *MAD2L1BP* 2 siRNA duplexes targeting the 3′-UTR were used at 40 nM each (sense: 5′-CAACAUCUCUUUGAAUCAAAGGUTG-3′ and 5′-AGAGCUUACAUCAGAAUCGAGCUTT-3′). DsiRNA targeting luciferase (*GL2*) was used as negative control. siRNA-transfected cells were grown for 12–24 hours prior to cell cycle synchronization. For arrest at the G_1_/S boundary, cells were treated with 2 mM thymidine (MilliporeSigma) for 18–20 hours. G_2_ arrest was achieved by addition of RO3306 (Santa Cruz Biotechnology, 10 μM) 6 hours after thymidine release. After 6 hours, RO3306-treated cells were synchronously released from G_2_ into mitosis by 5 cycles of medium exchange. For SAC override, Taxol-arrested HeLaK cells were harvested by shake-off and released for the indicated times by replating into medium supplemented with aurora B inhibitor ZM447439 (Tocris Biosciences, Bio-Techne, 5 μM), Taxol (Tocris Biosciences, Bio-Techne, 0.2 μg/mL), and cycloheximide (MilliporeSigma, 30 μg/mL). Synchronization in prometaphase was done by addition of Taxol (0.2 μg/mL) for 6 hours (presynchronized cells released from thymidine block 6 hours earlier) or 14 hours (asynchronous cells). To introduce a single DSB by I-SceI in G2, U2OS reporter cells released from a single thymidine block for 6 hours were treated for 48 hours with 1 μM 4-hydroxytamoxifen (MilliporeSigma) and, where indicated, 10 μM SCR7 (MedChemExpress) or carrier solvent. For genome-wide DSB infliction, asynchronous cell cultures were supplemented with DRB (LC Laboratories, 0.5 μM) for 2 hours.

### Flow cytometry

To measure the amount of GFP fluorescence in the U2OS reporter lines (18, 19), cells were trypsinized 48 hours after DSB infliction, resuspended in fresh media, mixed 2:1 with 10% formaldehyde, and immediately analyzed as described (18). Samples were analyzed on a Cytomix FC 500 (Beckman Coulter) using FL1 signal detector for FITC (GFP-positive cells) and FL2 for propidium iodide (PI) and counting at least 10,000 single cells per condition. Flow cytometry of PI-stained cells was done as described (56).

### In vitro transcription-translation

FLAG_3_-TEV_2_-p31^comet^ variants were in vitro expressed by combining a corresponding pCS2 expression plasmid with SP6 RNA polymerase-supplemented rabbit reticulocyte lysate (TNT SP6 Coupled Transcription/Translation Quick System from Promega) according to the manufacturers’ instructions. In vitro transcription-translation (5 μL) was supplemented with TEV protease in TEV cleavage buffer (10 mM HEPES-KOH pH 7.7, 50 mM NaCl, 25 mM NaF, 1 mM EGTA, 20% glycerol) and incubated for 20 minutes at room temperature. Reactions were stopped by boiling in SDS sample buffer.

### WES of patient 1a and 1b JGCT DNA and analysis for mutational signatures

DNA of tumor-normal pairs from both siblings was isolated from FFPE slides and whole blood, respectively. Sequencing libraries were prepared using the Twist Library Preparation Enzymatic Fragmentation Kit and the Twist Hybridization and Wash Kit (Twist Bioscience) with an optimized manufacturer protocol. Paired-end WES was performed on an Illumina NovaSeq 6000 system. Detection of germline and somatic SNVs, small insertions/deletions (indels), and copy number variants as well as calculation of TMB and MSI were performed as described previously (57–59). Briefly, we used the megSAP NGS analysis pipeline (https://github.com/imgag/megSAP, version 2021_12) and the ngs-bits package (https://github.com/imgag/ngs-bits, version 2022_04). Sequencing reads were aligned to the human reference genome (GRCh38) with BWA-MEM (60). Somatic SNVs and indels were called using Strelka2 (61) and annotated with Ensembl VEP (62). To obtain high-confidence somatic variant lists, variants were required to have sufficient depth of coverage of at least 20× and an allele frequency of at least 5%. Somatic copy number alterations were detected using ClinCNV (63). Genes affected by mutations were additionally annotated with oncogenicity information from the Network of Cancer Genes. TMB was calculated based on the number of variants in the exome per megabase. MSI status was evaluated with MANTIS (64). Text reports summarizing all relevant findings (oncogenic variants and additional biomarkers) were generated with GSvar (https://github.com/imgag/ngs-bits, version 2022_04).

We identified known mutational signatures in the 2 tumor samples using SigProfilerExtractor (https://github.com/AlexandrovLab/SigProfilerExtractor) on the somatic mutations identified by WES. The approach is to find the best way to explain observed somatic mutations (M) by known mutational signatures (S) and their activity (extent of contribution to the mutation profile A). Signature detection is an unsupervised machine learning approach and relies on matrix decomposition to find optimal solutions for S and A: observed somatic mutations M = mutational signatures S × activity of signatures A.

The observed distribution of nucleotide changes is decomposed into known signatures (https://cancer.sanger.ac.uk/signatures/sbs/), using an iterative approach of adding and removing reference signatures (nonnegative least square algorithm with add-remove steps). Added reference signatures are kept if they, in combination with signatures added in previous iterations, improve the reconstruction of the observed distribution of nucleotide changes. As a result, the method returns the mutational signatures and their relative contributions that best explain the observed nucleotide changes.

### Statistics

Simple comparisons were performed by unpaired, 2-tailed Student’s *t* test. The data were analyzed using Prism version 9.5.1. (GraphPad Software). All statistical tests were performed at the 5% significance level or were more stringent as indicated.

### Study approval

The study was approved by the Institutional Review Boards of the Ethics Committees of the University Hospital of Cologne; the National Research Centre, Cairo; and King Fahad Medical City. The parents provided written informed consent to participate in the study for themselves and for their children, including genetic investigations and publication of facial images.

### Data availability

WES data from the 3 patients reported herein are available in a public repository, NCBI BioSample, https://www.ncbi.nlm.nih.gov/bioproject/PRJNA915310 (accession PRJNA915310).

## Author contributions

GMHAS, MME, SAT, and EAF identified the patients and carried out their clinical and cytogenetic characterization. SH, EG, SK, JW, DTS, ASP, HT, PN, BSB, MRT, IBB, CS, AG, SO, FH, HR, DS, ABA, PB, JNP, RP, AH, CG, DS, UZ, OS, SS, and HJB designed and/or performed experiments and/or performed data analysis. OS directed the functional analyses in cell culture models. SS directed the functional characterizations of patients’ fibroblasts. HJB directed the human genetic analyses. OS, SS, and HJB jointly supervised the project and wrote the manuscript with input from GMHAS, SH, EG, SK, JW, ASP, MME, HT, PN, BSB, MRT, IBB, CS, AG, SO, FH, HR, MSAH, IH, AGM, DTS, ABA, PB, JNP, RP, AH, CG, DS, UZ, SAT, EAF, OS, SS, and HJB, who approved the final version.

## Supplementary Material

Supplemental data

Supporting data values

## Figures and Tables

**Table 1:**
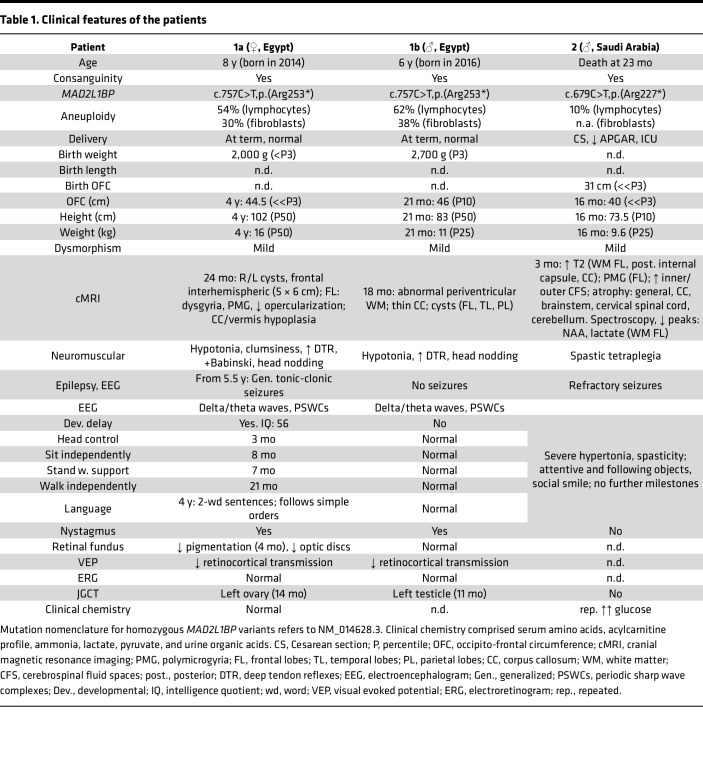
Clinical features of the patients

## Version 1 10/05/2023

In-Press Preview

## Version 2 11/22/2023

Electronic publication

## References

[1] Hassold T (2007). The origin of human aneuploidy: where we have been, where we are going. Hum Mol Genet.

[2] Vasudevan A (2021). Aneuploidy as a promoter and suppressor of malignant growth. Nat Rev Cancer.

[3] Kops GJ (2005). On the road to cancer: aneuploidy and the mitotic checkpoint. Nat Rev Cancer.

[4] Xia G (2004). Conformation-specific binding of p31(comet) antagonizes the function of Mad2 in the spindle checkpoint. EMBO J.

[5] Eytan E (2014). Disassembly of mitotic checkpoint complexes by the joint action of the AAA-ATPase TRIP13 and p31(comet). Proc Natl Acad Sci U S A.

[6] Carvalhal S (2022). Biallelic BUB1 mutations cause microcephaly, developmental delay, and variable effects on cohesion and chromosome segregation. Sci Adv.

[7] Hanks S (2004). Constitutional aneuploidy and cancer predisposition caused by biallelic mutations in BUB1B. Nat Genet.

[8] Yost S (2017). Biallelic TRIP13 mutations predispose to Wilms tumor and chromosome missegregation. Nat Genet.

[9] Replogle JM (2020). Aneuploidy increases resistance to chemotherapeutics by antagonizing cell division. Proc Natl Acad Sci U S A.

[10] Alfieri C (2018). Mechanism for remodelling of the cell cycle checkpoint protein MAD2 by the ATPase TRIP13. Nature.

[11] Hellmuth S (2020). Securin-independent regulation of separase by checkpoint-induced shugoshin-MAD2. Nature.

[12] Ma HT, Poon RYC (2016). TRIP13 regulates both the activation and inactivation of the spindle-assembly checkpoint. Cell Rep.

[13] Weaver BA, Cleveland DW (2005). Decoding the links between mitosis, cancer, and chemotherapy: the mitotic checkpoint, adaptation, and cell death. Cancer Cell.

[14] Gascoigne KE, Taylor SS (2008). Cancer cells display profound intra- and interline variation following prolonged exposure to antimitotic drugs. Cancer Cell.

[15] Shi J (2008). Cell type variation in responses to antimitotic drugs that target microtubules and kinesin-5. Cancer Res.

[16] Sarangi P (2020). p31^comet^ promotes homologous recombination by inactivating REV7 through the TRIP13 ATPase. Proc Natl Acad Sci U S A.

[17] Clairmont CS (2020). TRIP13 regulates DNA repair pathway choice through REV7 conformational change. Nat Cell Biol.

[18] Gunn A, Stark JM (2012). I-SceI-based assays to examine distinct repair outcomes of mammalian chromosomal double strand breaks. Methods Mol Biol.

[19] Pierce AJ (1999). XRCC3 promotes homology-directed repair of DNA damage in mammalian cells. Genes Dev.

[20] Hellmuth S (2018). Local activation of mammalian separase in interphase promotes double-strand break repair and prevents oncogenic transformation. EMBO J.

[21] Stelzl U (2005). A human protein-protein interaction network: a resource for annotating the proteome. Cell.

[22] Garcia-Castillo H (2008). Clinical and genetic heterogeneity in patients with mosaic variegated aneuploidy: delineation of clinical subtypes. Am J Med Genet A.

[23] Jacquemont S (2002). High risk of malignancy in mosaic variegated aneuploidy syndrome. Am J Med Genet.

[24] Callier P (2005). Microcephaly is not mandatory for the diagnosis of mosaic variegated aneuploidy syndrome. Am J Med Genet A.

[25] Snape K (2011). Mutations in CEP57 cause mosaic variegated aneuploidy syndrome. Nat Genet.

[26] De Wolf B (2021). Chromosomal instability by mutations in the novel minor spliceosome component CENATAC. EMBO J.

[27] Villarroya-Beltri C (2022). Biallelic germline mutations in MAD1L1 induce a syndrome of aneuploidy with high tumor susceptibility. Sci Adv.

[28] Grange LJ (2022). Pathogenic variants in SLF2 and SMC5 cause segmented chromosomes and mosaic variegated hyperploidy. Nat Commun.

[29] Chen OJ (2022). Germline missense variants in CDC20 result in aberrant mitotic progression and familial cancer. Cancer Res.

[30] Sotillo R (2007). Mad2 overexpression promotes aneuploidy and tumorigenesis in mice. Cancer Cell.

[31] Goudie C (2018). Paediatric ovarian tumours and their associated cancer susceptibility syndromes. J Med Genet.

[32] Palles C (2022). Germline MBD4 deficiency causes a multi-tumor predisposition syndrome. Am J Hum Genet.

[33] Plon SE (2008). Multiple tumors in a child with germ-line mutations in TP53 and PTEN. N Engl J Med.

[34] Nogales FF (2004). Multifocal intrafollicular granulosa cell tumor of the ovary associated with an unusual germline p53 mutation. Mod Pathol.

[35] Gu Y (2022). Evolutionary dynamics and molecular mechanisms of HORMA domain protein signaling. Annu Rev Biochem.

[36] Taylor AM (2018). Genomic and functional approaches to understanding cancer aneuploidy. Cancer Cell.

[37] Bronder D (2021). TP53 loss initiates chromosomal instability in fallopian tube epithelial cells. Dis Model Mech.

[38] Schvartzman JM (2011). Mad2 is a critical mediator of the chromosome instability observed upon Rb and p53 pathway inhibition. Cancer Cell.

[39] Choi E (2016). Mitotic checkpoint regulators control insulin signaling and metabolic homeostasis. Cell.

[40] Rio Frio T (2010). Homozygous BUB1B mutation and susceptibility to gastrointestinal neoplasia. N Engl J Med.

[41] Ruschendorf F, Nurnberg P (2005). ALOHOMORA: a tool for linkage analysis using 10K SNP array data. Bioinformatics.

[42] Abecasis GR (2001). GRR: graphical representation of relationship errors. Bioinformatics.

[43] O’Connell JR, Weeks DE (1998). PedCheck: a program for identification of genotype incompatibilities in linkage analysis. Am J Hum Genet.

[44] Abecasis GR (2002). Merlin--rapid analysis of dense genetic maps using sparse gene flow trees. Nat Genet.

[45] Thiele H, Nurnberg P (2005). HaploPainter: a tool for drawing pedigrees with complex haplotypes. Bioinformatics.

[46] Liu X (2016). dbNSFP v3.0: a one-stop database of functional predictions and annotations for human nonsynonymous and splice-site SNVs. Hum Mutat.

[47] Elsayed SM (2015). Non-manifesting AHI1 truncations indicate localized loss-of-function tolerance in a severe Mendelian disease gene. Hum Mol Genet.

[48] Beck BB (2014). Mutation of POC1B in a severe syndromic retinal ciliopathy. Hum Mutat.

[49] Lek M (2016). Analysis of protein-coding genetic variation in 60,706 humans. Nature.

[50] Lelieveld SH (2016). Meta-analysis of 2,104 trios provides support for 10 new genes for intellectual disability. Nat Neurosci.

[51] Shah SP (2009). Mutation of FOXL2 in granulosa-cell tumors of the ovary. N Engl J Med.

[52] Baillard P (2021). Rare DICER1 and absent FOXL2 mutations characterize ovarian juvenile granulosa cell tumors. Am J Surg Pathol.

[53] Vangipuram M (2013). Skin punch biopsy explant culture for derivation of primary human fibroblasts. J Vis Exp.

[54] Hauser F (2020). A non-invasive diagnostic assay for rapid detection and characterization of aberrant mRNA-splicing by nonsense mediated decay inhibition. Mol Genet Metab.

[55] Stemmann O (2001). Dual inhibition of sister chromatid separation at metaphase. Cell.

[56] Hellmuth S (2014). PP2A delays APC/C-dependent degradation of separase-associated but not free securin. EMBO J.

[57] Forschner A (2020). MDM2, MDM4 and EGFR amplifications and hyperprogression in metastatic acral and mucosal melanoma. Cancers (Basel).

[58] Hilke FJ (2020). Distinct mutation patterns reveal melanoma subtypes and influence immunotherapy response in advanced melanoma patients. Cancers (Basel).

[59] Eckardt J (2022). TMB and BRAF mutation status are independent predictive factors in high-risk melanoma patients with adjuvant anti-PD-1 therapy. J Cancer Res Clin Oncol.

[60] Li H, Durbin R (2009). Fast and accurate short read alignment with Burrows-Wheeler transform. Bioinformatics.

[61] Kim S (2018). Strelka2: fast and accurate calling of germline and somatic variants. Nat Methods.

[62] McLaren W (2016). The Ensembl variant effect predictor. Genome Biol.

[64] Kautto EA (2017). Performance evaluation for rapid detection of pan-cancer microsatellite instability with MANTIS. Oncotarget.

[65] Murakumo Y (2000). A human REV7 homolog that interacts with the polymerase zeta catalytic subunit hREV3 and the spindle assembly checkpoint protein hMAD2. J Biol Chem.

