# Supplementary material for: Biallelic MAD2L1BP (p31comet) mutation is associated with mosaic aneuploidy and juvenile granulosa cell tumors
Source: JCI Insight. 2023 Nov 22;8(22):e170079. doi: 10.1172/jci.insight.170079 (PMC10721328; doi:10.1172/jci.insight.170079)
Supplement: Supplemental data [file jciinsight-8-170079-s204.pdf]

SUPPLEMENTAL MATERIAL

Biallelic *MAD2L1BP* (p31^{comet}) mutation is associated with mosaic aneuploidy and juvenile granulosa cell tumors

Ghada M. H. Abdel-Salam[1], Susanne Hellmuth[2], Elise Gradhand[3], Stephan Käseberg[4], Jennifer Winter[4], Ann-Sophie Pabst[4], Maha M. Eid[5], Holger Thiele[6], Peter Nürnberg[6,7,8], Birgit S. Budde[6], Mohammad Reza Toliat[6], Ines B. Brecht[9], Christopher Schroeder[10], Axel Gschwind[10], Stephan Ossowski[10], Friederike Häuser[11], Heidi Rossmann[11], Mohamed S Abdel-Hamid[12], Ibrahim Hegazy[1], Ahmed G. Mohamed[13], Dominik T. Schneider[14], Aida Bertoli-Avella[15], Peter Bauer[15], Jillian N. Pearring[16,17], Rolph Pfundt[18], Alexander Hoischen[18,19], Christian Gilissen[18], Dennis Strand[20], Ulrich Zechner[4,21], Soha A. Tashkandi[22], Eissa A. Faqeih[23], Olaf Stemmann[2], Susanne Strand[20], Hanno J. Bolz[21,24]

[1]Department of Clinical Genetics, Human Genetics and Genome Research Institute, National Research Centre, Cairo, Egypt

[2]Chair of Genetics, University of Bayreuth, Bayreuth, Germany

[3]Senckenberg Institute of Pathology, University Hospital Frankfurt, Frankfurt, Germany

[4]Institute of Human Genetics, University Medical Center Mainz, Mainz, Germany

[5]Human Cytogenetics Department, Human Genetics and Genome Research Institute, National Research Centre, Cairo, Egypt

[6]Cologne Center for Genomics, University Hospital of Cologne, University of Cologne, Cologne, Germany

[7]Center for Molecular Medicine Cologne, University Hospital of Cologne, University of Cologne, Cologne, Germany

[8]Cologne Excellence Cluster on Cellular Stress Responses in Aging-Associated Diseases (CECAD), University of Cologne, Cologne, Germany

[9]Paediatric Haematology/Oncology, Department of Paediatrics, University Hospital Tübingen, Germany

[10]Institute of Medical Genetics and Applied Genomics, Eberhard-Karls University, Tübingen, Germany

[11]Institute of Clinical Chemistry and Laboratory Medicine, University Medical Center Mainz, Mainz, Germany

[12]Medical Molecular Department, Human Genetics and Genome Research Institute, National Research Centre, Cairo, Egypt

[13]Pediatrics Department, Faculty of Medicine, Cairo University, Cairo, Egypt

[14]Clinic of Pediatrics, University Witten/Herdecke, Dortmund, Germany

[15]CENTOGENE GmbH, Rostock, Germany

[16]Department of Ophthalmology, University of Michigan, Ann Arbor, Michigan

[17]Department of Cell and Developmental Biology, University of Michigan, Ann Arbor, Michigan

[18]Department of Human Genetics and Radboud Institute for Molecular Life Sciences, Radboud University Medical Centre, Nijmegen, The Netherlands

[19]Department of Internal Medicine, Radboud University Medical Center, Nijmegen, The Netherlands

[20]Department of Internal Medicine I, University Medical Center Mainz, Mainz, Germany

[21]Senckenberg Centre for Human Genetics, Frankfurt am Main, Germany

[22]Cytogenetics Laboratory, Pathology and Clinical Laboratory Medicine Administration (PCLMA), King Fahad Medical City, Second Central Healthcare Cluster (C2), Riyadh, Kingdom of Saudi Arabia

[23]Section of Medical Genetics, Children's Specialist Hospital, King Fahad Medical City, Riyadh, Kingdom of Saudi Arabia

[24]Institute of Human Genetics, University Hospital of Cologne, University of Cologne, Cologne, Germany

GMHAS and SH contributed equally to this work.

OS, SS and HJB contributed equally to this work.

Correspondence to:

Hanno J. Bolz, MD. Present address: Bioscientia Human Genetics, Institute for Medical Diagnostics, Konrad-Adenauer-Str. 17, 55218 Ingelheim, Germany; Institute of Human Genetics, University Hospital of Cologne, Kerpener Str. 34, Cologne, Germany. Phone: +49 6132781206. Email: hanno.bolz@icloud.com

Conflict-of-interest statement

The authors have declared that no conflict of interest exists.

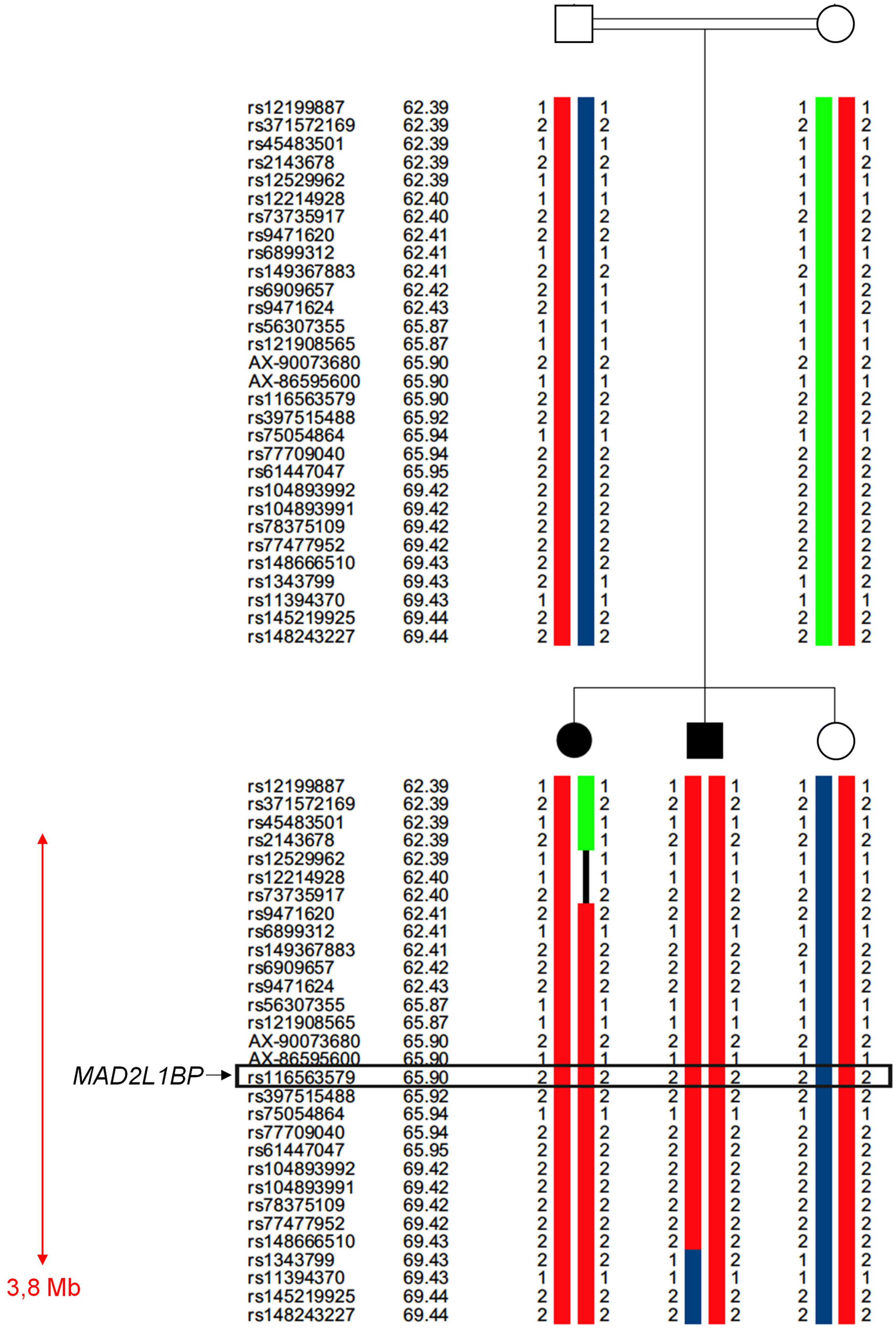

SUPPLEMENTAL FIGURE 1

Haplotype of the *MAD2L1BP* region on chromosome 6.

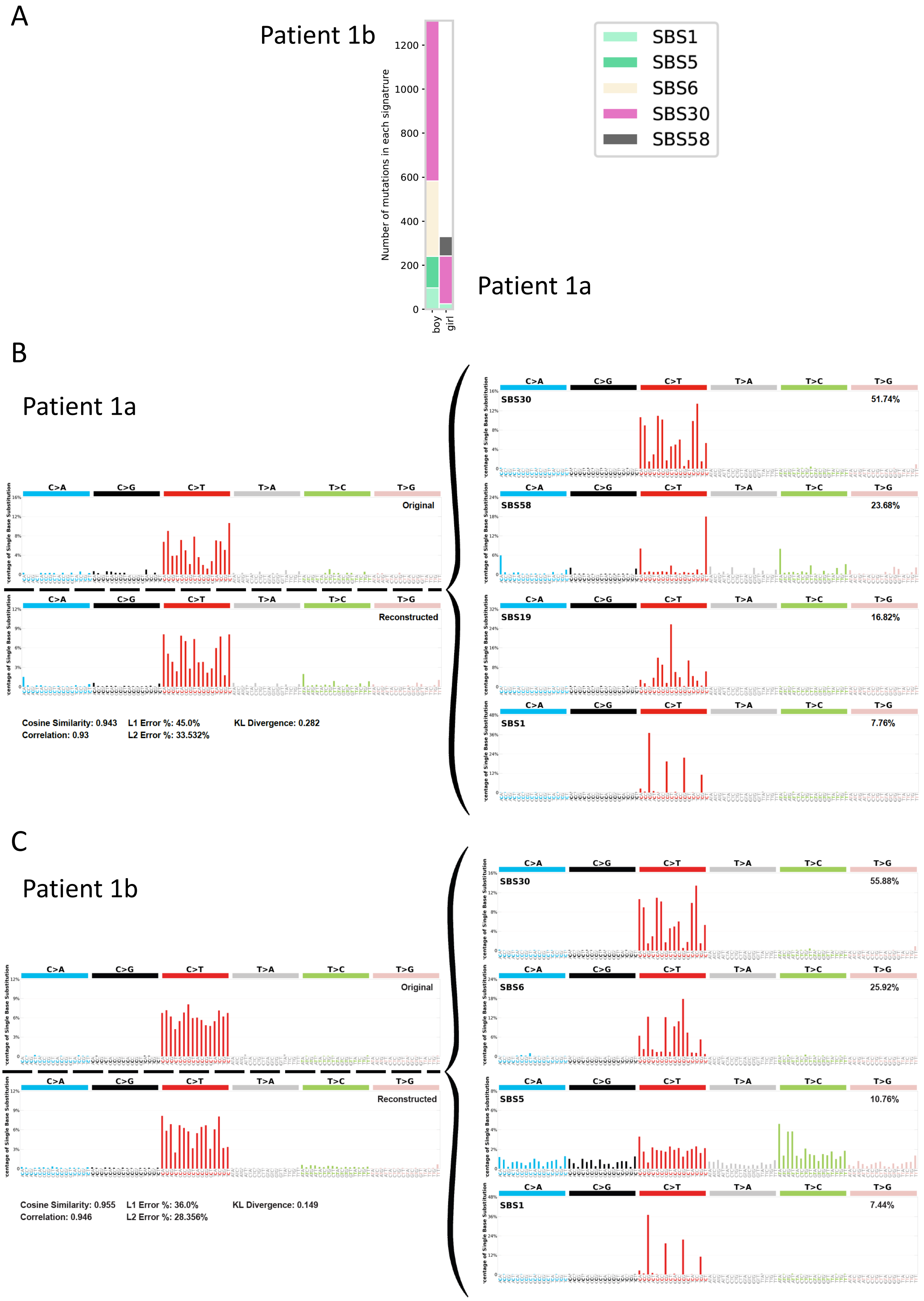

SUPPLEMENTAL FIGURE 2

Mutational signature using unfiltered somatic variants (MAF ≥ 2.5%, no quality threshold). (**A**) Relative contribution of identified signatures in the two samples. (**B**) Signature decomposition for the sample of Patient 1a. (**C**) Signature decomposition for the sample of Patient 1b. Both mutational profiles are dominated by SBS30 (putative FFPE-damage signature).

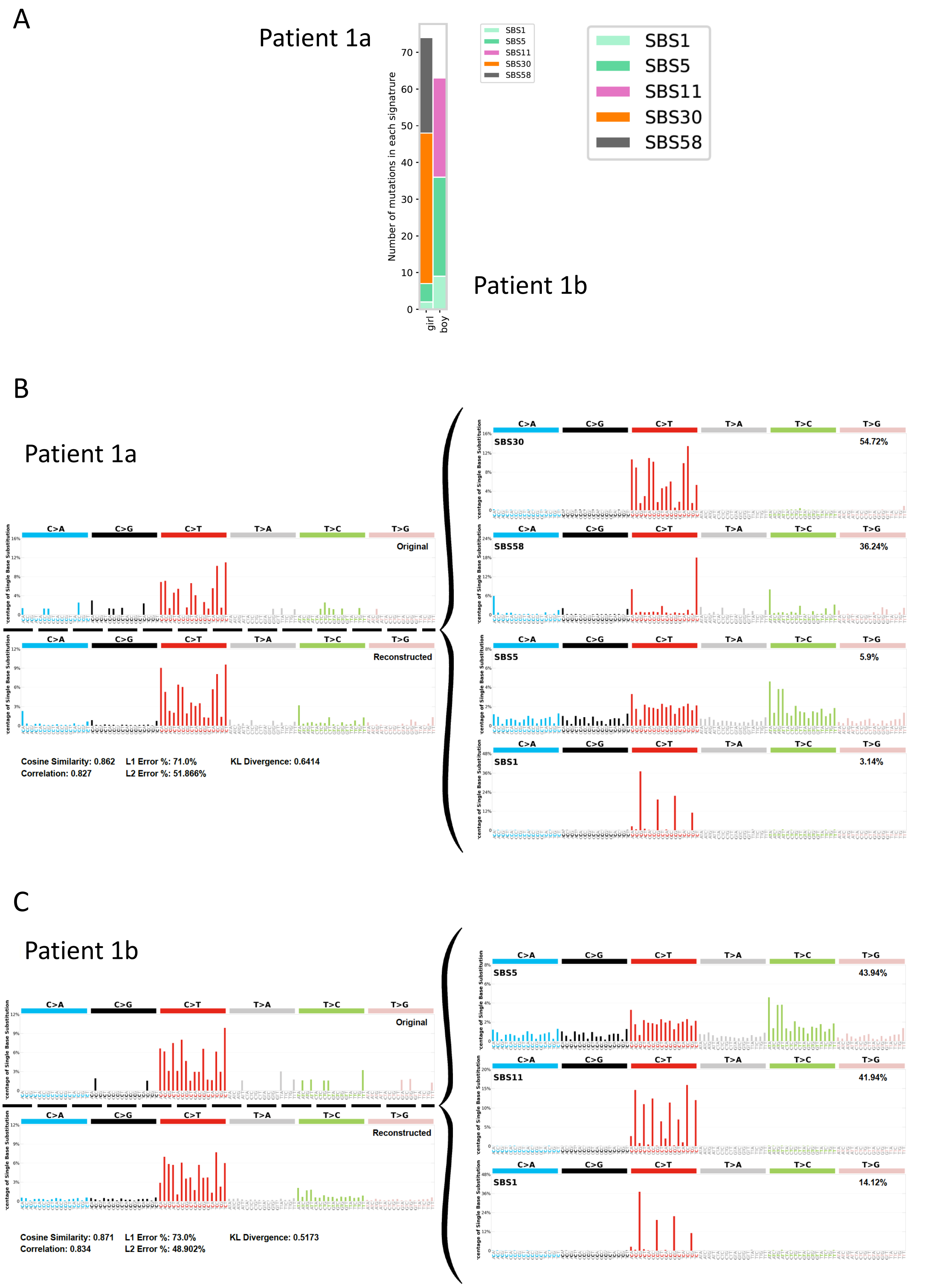

SUPPLEMENTAL FIGURE 3

Mutational signature using high quality somatic variants with MAF ≥5%. (**A**) Relative contribution of identified signatures in the two samples. (**B**) Signature decomposition for the sample of Patient 1a. (**C**) Signature decomposition for the sample of Patient 1b. Quality filtering of somatic mutations revealed a contribution of signatures SBS5 and SBS11 in Patient 1b.

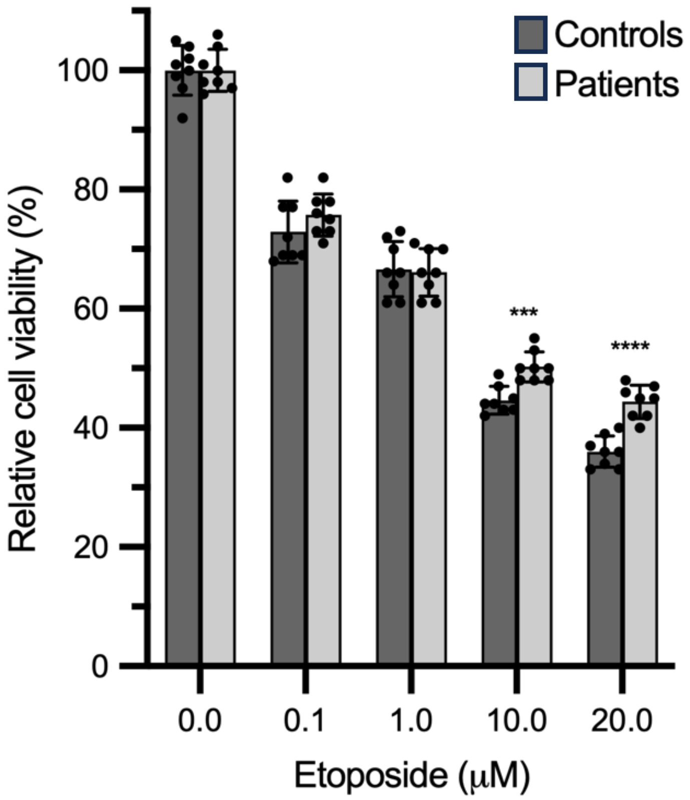

SUPPLEMENTAL FIGURE 4

Dose response of etoposide. Cell viability of fibroblasts from controls and patients were treated for 72h with the indicated concentrations of etoposide normalized to DMSO vehicle. Error bars represent the s.d. (n = 4). Data were analyzed by two-tailed, unpaired Students t-test (****p<0.0001, ***p<0.001; **p<0.01; *p<0.05). Each experiment was repeated 3 times.

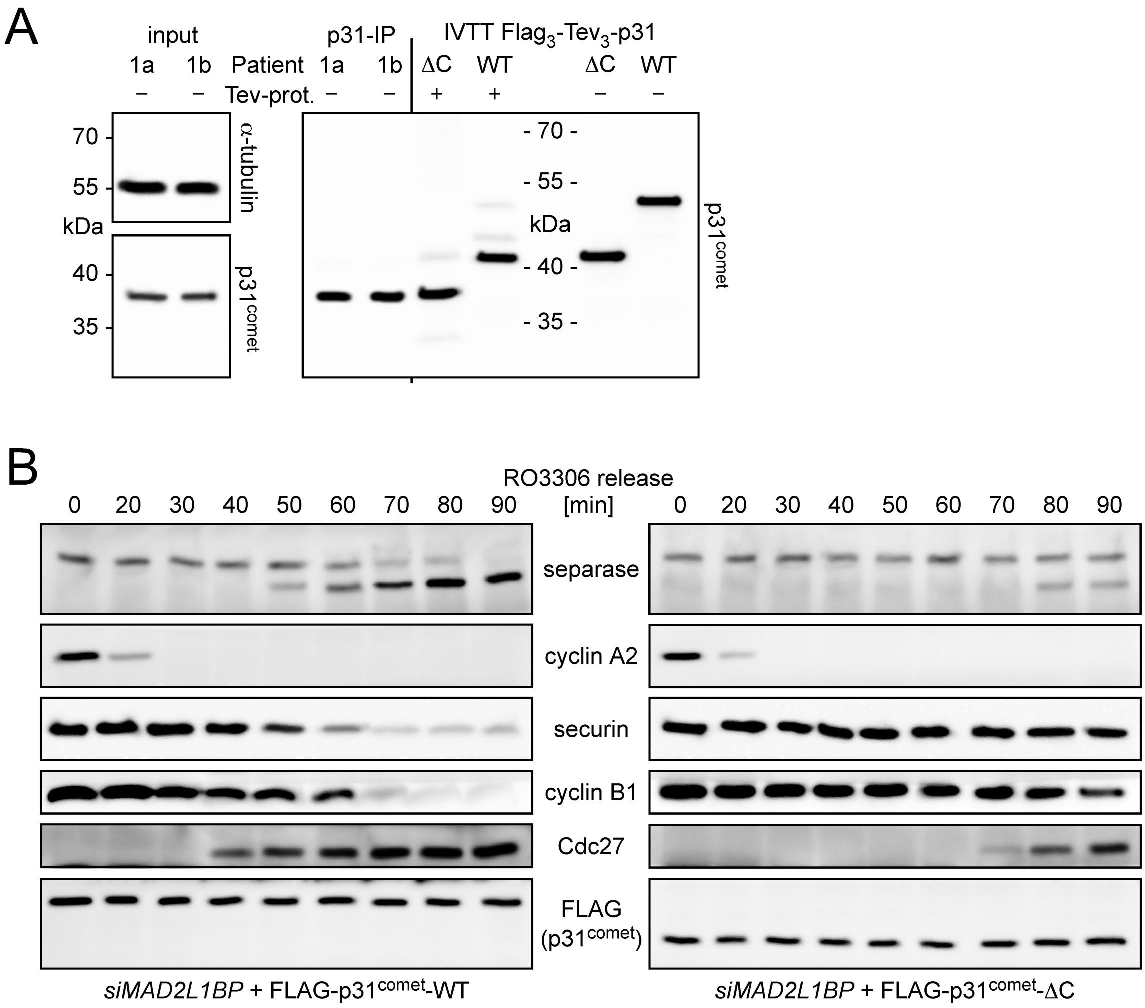

SUPPLEMENTAL FIGURE 5

Characterization of recombinant p31^{comet}-ΔC. (**A**) Recombinant p31^{comet}-ΔC and patient derived, endogenous p31^{comet} exhibit the same molecular weight in SDS-PAGE. Endogenous p31^{comet} from asynchronously growing, patient-derived fibroblasts was immunoprecipitated and analyzed by SDS-PAGE and immunoblotting side-by-side with the indicated *in vitro* translated (IVT) p31^{comet} variants, from which the $FLAG_3$-Tev_2-tag had (or had not) been removed by treatment with TEV protease. (**B**) Cells that rely on p31^{comet}-ΔC progress slowly through mitosis. HeLaK cells transfected to replace endogenous p31^{comet} by the indicated $FLAG_3$-Tev_2-tagged variants were synchronously released from a RO3306-mediated G2-arrest and analyzed by flow cytometry (Figure 6B) and time-resolved immunoblotting. Note that cyclin A2, a SAC-independent APC/C substrate, is degraded with normal kinetics in p31^{comet}-ΔC expressing cells indicating that entry into mitosis is not delayed. (Note also that Cdc27 is hyperphosphorylated in early mitosis and becomes visible as a sharp band only upon dephosphorylation in late M-phase.)

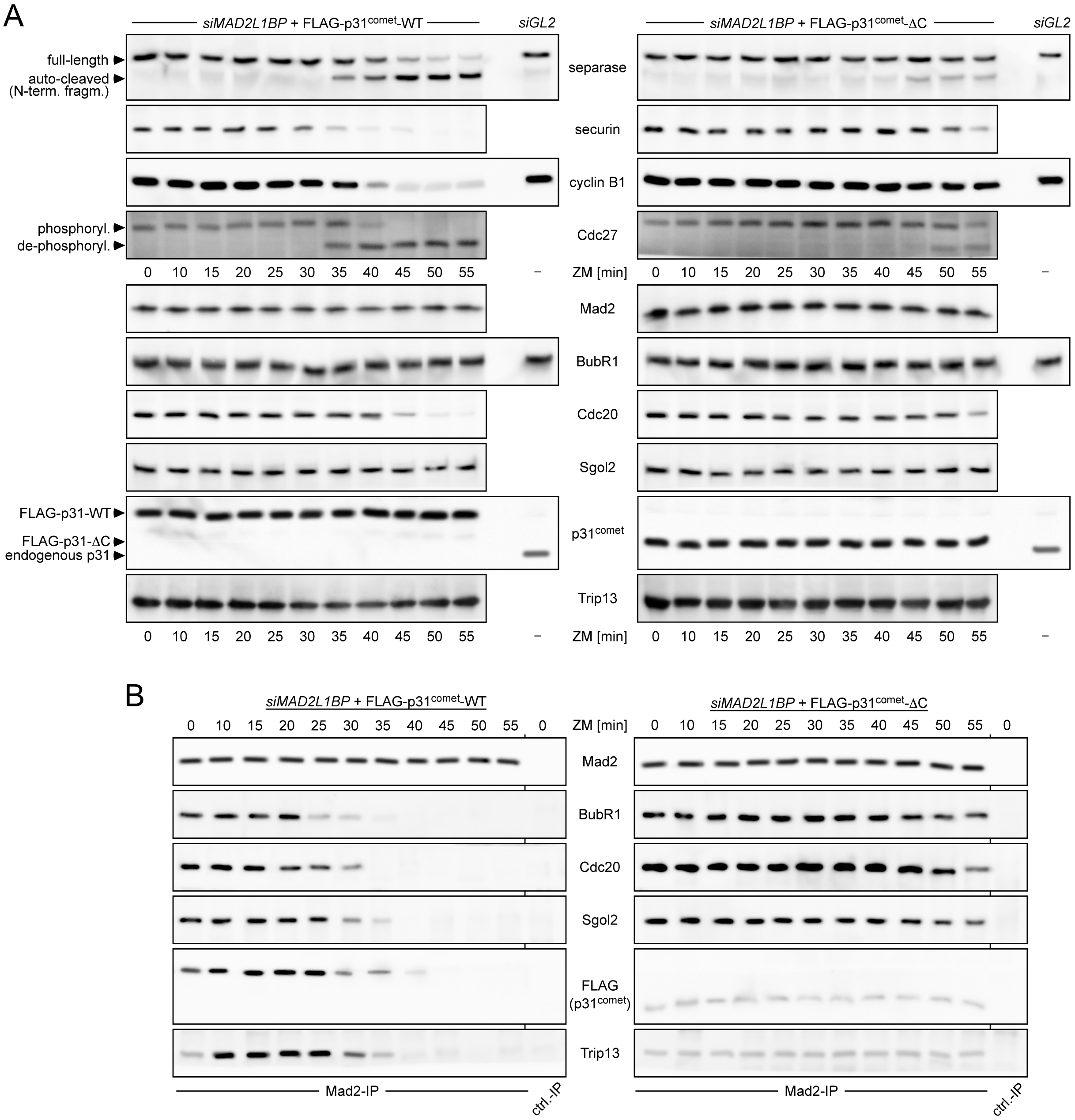

SUPPLEMENTAL FIGURE 6

Functional characterization of p31^{comet}-ΔC in mitosis. (**A**) Replacing endogenous p31^{comet} by p31^{comet}-ΔC greatly prolongs metaphase. HeLaK cells transfected to replace endogenous p31^{comet} by the indicated FLAG$_3$-Tev$_2$-tagged variants were synchronously released from a taxol-mediated prometaphase arrest by the aurora B inhibitor ZM 447439 (ZM) and analyzed by time-resolved immunoblotting using the indicated antibodies. (**B**) p31^{comet}-ΔC cannot support the disassembly of SAC-induced Mad2 complexes. Cells from (A) were subjected to time-resolved Mad2-IPs followed by immunoblotting using the indicated antibodies.

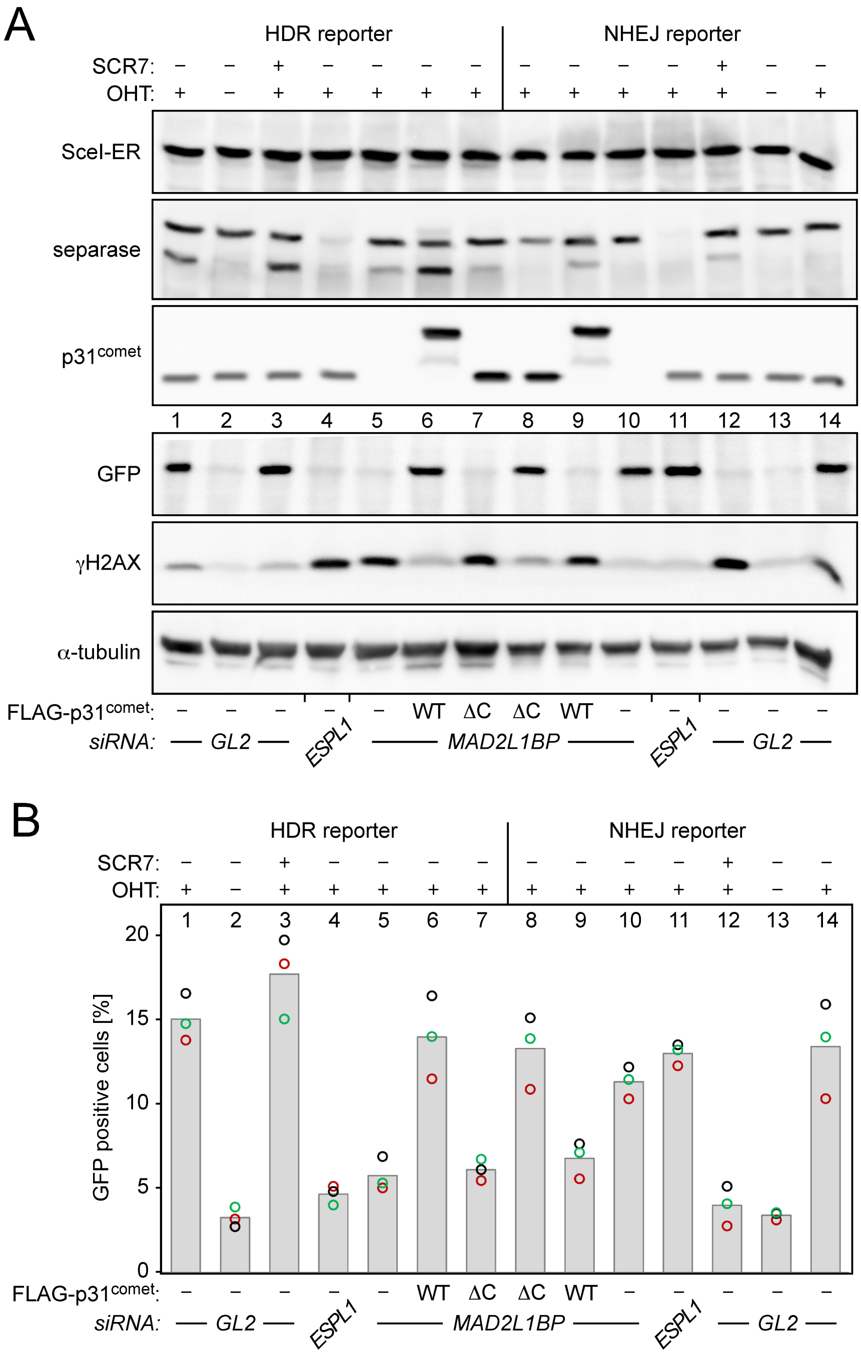

SUPPLEMENTAL FIGURE 7

C-terminally truncated p31^{comet} cannot support homology-directed repair of DNA double strand breaks. (**A**) U2OS DR-GFP (HDR reporter) and U2OS EJ5-GFP (NHEJ reporter) cells transiently expressing HA- and estrogen receptor-tagged I-SceI (HA-ER-I-SceI) and Flag-tagged p31^{comet} variants were transfected with the indicated siRNAs, synchronized in G2-phase and supplemented - where indicated - with 4-hydroxytamoxifen (OHT) to direct the homing endonuclease into the nucleus and with ligase IV inhibitor SCR7 to block NHEJ. Two days later, cells were subjected to immunoblotting. (**B**) Samples from (A) were also analyzed by flow cytometry to determine the percentage of GFP-positive cells. Shown are averages (bars) of three independent experiments (dots). Note that overexpression of p31^{comet}-WT has a suppressive effect on NHEJ. *SEP, SEPARASE*, positive control for a protein required for HDR.

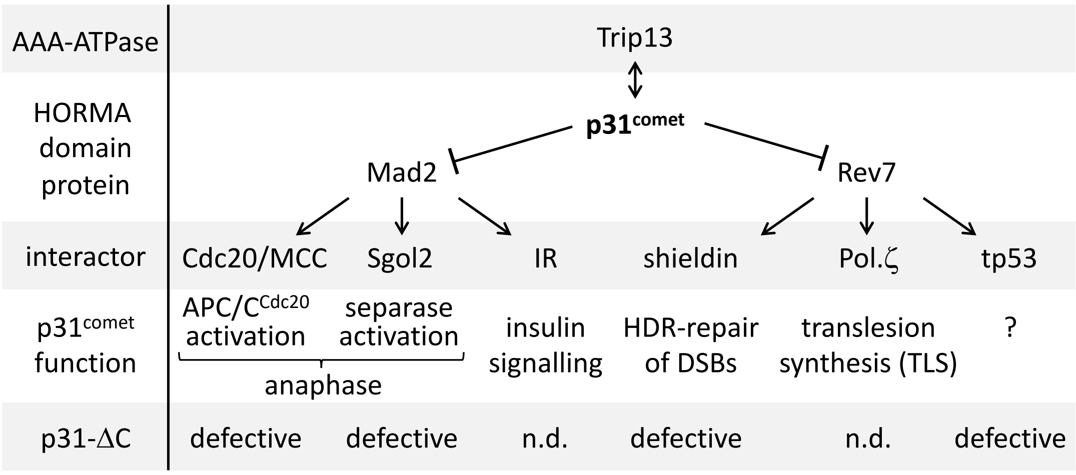

SUPPLEMENTAL FIGURE 8

Summary of p31^{comet} interactions and functions. p31^{comet}-ΔC is unable to support onset of anaphase or homology directed repair of DSBs. IR, insulin receptor; n.d., not determined.

Gene	Chr (Mb)	Transcript	Variant, (DNA)	Variant, (protein)	Function Monogenic disease	MAF (%) rs number	homo/ hemi (gnomAD)	Pathogenicity predictions	HBD region
ZNF654	3 88,139	NM_001350134.1	c.1972G>A	p.(Val658Met)	Zinc finger protein; transcriptional activation?	0.000306 rs138407348	1	0/11	yes
EPHA3	3 89,209	NM_182644.2	c.275C>T	p.(Ala92Val)	Receptor tyrosine kinase; regulates cell-cell adhesion, cytoskeletal organization and cell migration. Role in cardiac cells migration and in the retinotectal mapping of neurons during development.	0.0000159 rs539750746	-	6/11	yes
HACD2	3 123,528	NM_198402.3	c.373G>A	p.(Val125Ile)	3-hydroxyacyl-CoA dehydratase 2; catalyzes dehydration in the conversion of long chain to very long chain fatty acids.	0.000161 rs202187144	-	2/11	yes
ZXDC	3 126,441	NM_025112.4	c.2246A>C	p.(Glu749Ala)	ZXD family zinc finger C	- -	-	0/11	yes
MAD2L1BP	6 43,640	NM_001003690.1	c.757C>T	p.(Arg253*)	Mosaic variegated aneuploidy (this study)	0.0000121 rs528509686	-	3/4	yes
RECQL4	8 144,511	NM_004260.3	c.3133G>A	p.(Ala1045Thr)	RecQ like helicase 4; biallelic variants may cause the skin disorder Rothmund-Thomson syndrome (RTS) [MIM:268400], RAPADILINO syndrome (RAPADILINOS) [MIM:266280] with radial and patellar hypo- or aplasia or Baller-Gerold syndrome (BGS) [MIM:218600], a craniosynostosis with radial defects.	0.00105 rs35348691	1	1/5	yes
			c.3337G>C	p.(Gly1113Arg)		0.000805 rs35101495	1	0/6	yes
ZNF618	9 114,049	NM_001318040.1	c.2124G>T	p.(Pro708=)	Belongs to krueppel C2H2-type zinc-finger protein family; transcriptional activation?	0.000226 rs368210659	-	0/3 (splice prediction)	yes
			c.2125G>T	p.(Val709Leu)		0.000229 rs527742445	-	2/11	yes
DEPDC4	12 100,267	NM_152317.3	c.62G>A	p.(Arg21His)	DEP domain containing 4; unknown function.	0.00000398 rs189364541	-	3/11	yes
MED13L	12 115,984	NM_015335.4	c.4459C>A	p.(Pro1487Thr)	Mediator complex subunit 13L; involved in early development of the heart and brain. Heterozygous pathogenic missense variants cause transposition of the great arteries, dextro-looped 1, (DTGA1) [MIM:608808]. Heterozygous loss-of-function variants cause mental retardation and distinctive facial features with or without cardiac defects (MRFACD) [MIM:616789].	0.000156 rs146112707	-	2/11	yes

Gene	Chr (Mb)	Transcript	Variant, (DNA)	Variant, (protein)	Function Monogenic disease	MAF (%) rs number	homo/ hemi (gnomAD)	Pathogenicity predictions	HBD region
FBXW8	12 117,028	NM_153348.2	c.1672G>A	p.(Ala558Thr)	Protein-ubiquitin ligase	0.000139 rs139429411	-	4/11	yes
B3GNT4	12 122,207	NM_030765.2	c.939G>T	p.(Arg313Ser)	Beta-1,3-N-acetylglucosaminyltransferase 4; involved in the biosynthesis of poly-N-acetyllactosamine sugar chains.	- -	-	0/10	yes
DDX55	12 123,602	NM_020936.2	c.8A>T	p.(His3Leu)	DEAD-box helicase 55; putative RNA helicases, involved in several nuclear processes?	- -	-	1/11	yes

SUPPLEMENTAL TABLE 1

Rare homozygous variants identified in WES and present in Patient 1a and Patient 1b, but not their healthy sister. Where applicable, diseases known to be associated with the respective genes are given. Red, disease gene, *MAD2L1BP*. Chr, chromosome; gnomAD, Genome Aggregation Database; homo, number of homozygous individuals annotated in gnomAD; rs, Reference SNP cluster ID. “Pathogenicity predictions”: Number of applicable pathogenicity prediction programmes that suggest functional impairment/pathogenicity.

Patient ID	Gender	JGCT	Age at diagnosis	WES	Tissue	*MAD2L1BP*	*DICER1* (tumor)	*FOXL2* (tumor)
1	F	ovary	12 ys.	+	normal	WT	N/A	N/A
2	F	ovary	7 ys.	+ (Tü)	normal	WT	N/A	N/A
3	F	ovary	15 ys.	+	normal	WT	N/A	N/A
4	F	ovary	11 mo.	+	normal + tumor	WT	WT	WT
5	F	ovary	13 ys.	+	normal	WT	N/A	N/A
6	F	ovary	N/A	+	tumor	WT	het c.4379C>G + c.5125G>A[a]	WT
7	F	ovary	4 mo.	+	tumor	WT	WT	WT
8	F	ovary	N/A	+	normal	WT	N/A	N/A
9	F	ovary	16 ys.	+ (Tü)	normal	WT	N/A	N/A
10	F	ovary	15 ys.	+ (Tü)	normal	WT	N/A	N/A
11	F	ovary	8 ys.	+	tumor	WT	WT	WT
12	F	ovary	2 ys.	+	tumor	WT	WT	WT
13	M	testis	1 mo.	+ (Tü)	normal	WT	N/A	N/A
14	F	ovary	22 ys.	+ (Tü)	normal	WT	N/A	N/A
15	F	ovary	10 ys.	+ (Tü)	normal	WT	N/A	N/A
16	F	ovary	13 ys.	+	tumor	het c.764C>T	WT	WT
17	F	ovary	16 ys.	+	normal	WT	N/A	N/A
18	F	ovary	13 ys.	+	normal	WT	N/A	N/A
19	F	ovary	N/A	+	tumor	WT	WT	WT[b]
20	F	ovary	11 ys.	+ (Tü)	normal	WT	N/A	N/A
21	F	ovary	2 ys.	+	tumor	WT	het c.4199A>G	WT
22	F	ovary	N/A	+	normal	WT	N/A	N/A
23	F	ovary	5 ys.	+	normal (Tü) + tumor	WT	WT	WT[b]

SUPPLEMENTAL TABLE 2

Results from *MAD2L1BP* mutation screening in patients with sporadic non-syndromic JGCT. [a], known somatic pathogenic variant, [b], coverage too low in some regions.

Allele frequency cut-off	Patient 1b, TMB	Patient 1b, count	Patient 1a, TMB	Patient 1a, count
2%	48.8	2,447	13.3	666
2.5%	30.6	1,534	8.5	427
3%	15.5	775	6.0	299
4%	4.7	233	3.9	194
5%	2.1	107	2.8	139

SUPPLEMENTAL TABLE 3

Somatic mutations and TMB (mutations per Mb).

Patient	TMB in mut/Mb (5%)	MSI score (MANTIS)
Patient 1b	2.1	0.236, MSI stable
Patient 1a	2.8	0.273, MSI stable

SUPPLEMENTAL TABLE 4

Complex biomarkers.

Gene	Variant	Type	Allele fraction	Description
NF1	c.4110+5G>A ENST00000358273.9	Splice region, intron	0,06	Possible splice defect, likely resulting in reduced gene expression or NMD

SUPPLEMENTAL TABLE 5

Oncogenic mutation in Patient 1a.
